# Different Strategies Affect Enzyme Packaging into Bacterial Outer Membrane Vesicles

**DOI:** 10.3390/bioengineering10050583

**Published:** 2023-05-11

**Authors:** Scott N. Dean, Meghna Thakur, Joseph R. Spangler, Aaron D. Smith, Sean P. Garin, Scott A. Walper, Gregory A. Ellis

**Affiliations:** 1 Center for Bio/Molecular Science and Engineering, Code 6900, U.S. Naval Research Laboratory, Washington, DC 20375, USA; 2 College of Science, George Mason University, Fairfax, VA 22030, USA

**Keywords:** outer membrane vesicles (OMVs), phosphotriesterase (PTE), diisopropyl fluorophosphatase (DFPase)

## Abstract

All Gram-negative bacteria are believed to produce outer membrane vesicles (OMVs), proteoliposomes shed from the outermost membrane. We previously separately engineered *E. coli* to produce and package two organophosphate (OP) hydrolyzing enzymes, phosphotriesterase (PTE) and diisopropylfluorophosphatase (DFPase), into secreted OMVs. From this work, we realized a need to thoroughly compare multiple packaging strategies to elicit design rules for this process, focused on (1) membrane anchors or periplasm-directing proteins (herein “anchors/directors”) and (2) the linkers connecting these to the cargo enzyme; both may affect enzyme cargo activity. Herein, we assessed six anchors/directors to load PTE and DFPase into OMVs: four membrane anchors, namely, lipopeptide Lpp’, SlyB, SLP, and OmpA, and two periplasm-directing proteins, namely, maltose-binding protein (MBP) and BtuF. To test the effect of linker length and rigidity, four different linkers were compared using the anchor Lpp’. Our results showed that PTE and DFPase were packaged with most anchors/directors to different degrees. For the Lpp’ anchor, increased packaging and activity corresponded to increased linker length. Our findings demonstrate that the selection of anchors/directors and linkers can greatly influence the packaging and bioactivity of enzymes loaded into OMVs, and these findings have the potential to be utilized for packaging other enzymes into OMVs.

## 1. Introduction

Extracellular vesicles are released from cells across all kingdoms of life and are involved in a multitude of biological processes. Bacterial outer membrane vesicles (OMVs) are spherical bilayered proteoliposomes ranging in size from 20 to 200 nm that bleb from the outermost membrane; are comprised of a variety of biomolecules such as proteins, lipids, and nucleic acids; and are involved in a variety of cellular functions such as cell–cell communication, transport of molecules, stress response, and infection [1]. While the proteinaceous content of OMVs is predominantly native membrane proteins [2], the contents of OMVs from pathogenic organisms have been demonstrated to include virulence factors [3] and lytic enzymes for lysing competitor cells [4]. On the other hand, the vesicles produced by probiotic organisms carry proteins associated with their probiotic effect and have been touted for their therapeutic potential [5,6,7]. Although the mechanism of OMV biogenesis is not well characterized, several reports have demonstrated the ability to package various non-native enzymes into vesicles, which beyond providing a platform of delivery, has also been shown to enhance their function under a variety of conditions [8].

Organophosphate (OP) hydrolases have garnered significant interest as bioremediation agents targeting organophosphates that are widely disseminated in the agriculture industry, leading to their accumulation at toxic levels in soil and water worldwide [9]. Examples include phosphotriesterase (PTE, e.g., from *Brevundimonas diminuta*) and diisopropylfluorophosphatase (DFPase, e.g., from *Loligo vlugaris*). OP exposures result in intoxication and neurological consequences and account for about 300,000 deaths per annum [10]. Spurred by this issue, there is a well-defined need for safe materials capable of fast bioremediation and personal decontamination, such as OP hydrolases, but without the relative fragility and narrow operational envelope of recombinant enzymes. One route for the creation of ruggedized enzymes is encapsulation in OMVs.

Previously, we showed the directed packaging of PTE into bacterial OMVs using a SpyCatcher/SpyTag (SC/ST) bioconjugation system [8,11]. In this system, the abundant membrane porin protein OmpA (truncated) acted as a membrane anchor and was attached to SpyTag [12]; PTE was in turn expressed with SpyCatcher to attach it to the OmpA anchor. The OMV-packaged PTE degraded the organophosphate paraoxon in a manner that was kinetically comparable to that of non-packaged recombinant PTE while reducing susceptibility to inactivation via multiple freeze–thaw cycles and lyophilization [8]. It could further maintain activity within environmental samples such as different water sources or solid surfaces such as glass, fabric, and painted metal surfaces [13]. More recently, we targeted OMV encapsulation of diisopropylfluorophosphatase (DFPase), another organophosphate hydrolase that can hydrolyze diisopropylfluorophosphate (DFP) and other chemical agents, using both a similar method and Lpp’ [14]. The OMV-encapsulated DFPase hydrolyzed both DFP and paraoxon, and packaging prevented loss of catalytic activity despite freeze-drying, extended storage at room temperature, and extreme temperatures of up to 80 °C. We note that beyond packaging in OMVs, various other methods have been reported with the objective of enhancing various features of PTE and DFPase including activity, stability, and/or half-life such as PEGylation [15] and mutation screening [16], among other alterations. In an analogous manner a wide range of parameters for optimization of paraoxon-degrading enzyme-OMV packaging can be adjusted, including improvements in packaging efficiency to obtain higher levels of enzyme per vesicle, increased OMV-production measured by OMV number, and improvement in the enzymatic activity on a per-vesicle basis.

**Figure 1:**
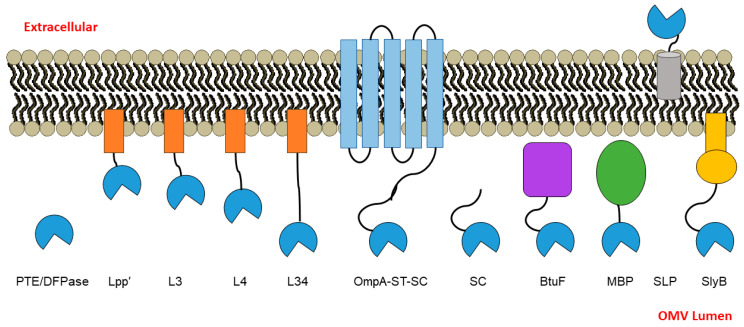
Schematic representation of the OMVs in this study. PTE or DFPase was expressed as a fusion protein with various anchors/directors, including to the anchor Lpp’ with increasing linker length (Lpp’, L3, L4, and L34), a truncated form of transmembrane porin protein (OmpA) as an anchor fused to SpyTag (ST) that binds PTE/DFPase fused to SpyCatcher (SC), PTE/DFPase-SC as a non-directed control, to periplasmic proteins as directors including maltose-binding protein (MBP) and vitamin B12-binding protein (BtuF), and to two additional lipoprotein anchors SLP and SlyB.

A key aspect in optimization of enzyme-OMV packaging is the ability to localize enzymes to the cell membrane so they are included in the released OMV. Since approximately 80 proteins have been identified as associated with *E. coli* OMVs [2], these proteins can be exploited for the design of anchors to target fusion proteins to desired locations of the OMV. Several groups have made use of the outer membrane lipoprotein Lpp’, while the membrane protein SlyB has been used to anchor nanoluciferase inside the lumen of OMVs [14,17,18,19]. Despite the high likelihood that specific fusion partners favorably impact stability and activity of the enzymes being packaged into OMVs, and that attachment to the membrane will also be impacted by linkers between the anchor and the conjugation method (e.g., SpyCatcher/SpyTag) or cargo, no systematic studies have been conducted to investigate this question. Here, we employed PTE and DFPase as our representative cargo enzymes into OMVs and compared packaging strategies using four different membrane-associated anchors (Lpp’, OmpA, SLP, and SlyB) and two different periplasmic-directing (but non-membrane associated) proteins (BtuF and MBP). Further, using one of these anchors (Lpp’), we also compared four different linkers between it and the enzyme cargo (see Figure 1 and Section 3 below for more detail). While each of the strategies led to packaging of PTE and DFPase in the OMVs and resulted in degradation of paraoxon, the extent of activity varied greatly between strategies. Importantly, these findings have the potential to be utilized with other enzymes in order to direct cargo into OMVs with increased packaging efficiencies and/or enhanced activities.

## 2. Materials and Methods

### 2.1. Bacterial Growth and OMV Purification

Starter cultures of *E. coli* BL21(DE3) were grown in lysogeny (LB) broth with shaking at 37 °C. BL21(DE3) with or without plasmids were maintained on LB agar (1.5% (*w*/*v*)) plates and grown in overnight cultures in the presence of no antibiotic, kanamycin (25 µg/mL), ampicillin (100 µg/mL), or chloramphenicol (25 µg/mL) for plasmid maintenance. For OMV production, 0.5 mL of overnight culture was used to inoculate 50 mL of terrific broth (TB) in baffled culture flasks. Cultures were grown for 3 h until an OD600 of 0.6−0.8 was reached. For pACYC184 AraC [PTE/DFPase]-SC-transformed BL21(DE3), arabinose was added to a final concentration of 0.2%; for all others, isopropyl β-1-D-thiogalactopyranoside (IPTG) was added to a final concentration of 0.5 mM, and the culture was allowed to grow for an additional 18 h at 37 °C.

Cultures were centrifuged twice at 7000× *g* for 15 min at 4 °C. The supernatant was filtered using 0.45 µm membrane to ensure cell removal, after which OMVs were pelleted at 29,000 rpm (≈150,000 g) in a Sorvall WX Ultra 90 centrifuge (ThermoFisher, Waltham, MA, USA) using an AH-629 rotor (ThermoFisher, Waltham, MA, USA) for 1.5 h at 4 °C in Ultra-Clear (25 × 89 mm) centrifuge tubes (Beckman Coulter, Brea, CA, USA). The culture media was decanted and the OMV pellet was resuspended in either 1 mL of N-cyclohexyl-2-aminoethanesulfonic acid (CHES) buffer (50 mM, pH 8.5) or phosphate-buffered saline (PBS) buffer and incubated overnight at 4 °C.

### 2.2. Genetic Contstructs

Genetic constructs containing the phosphotriesterase (PTE) gene from *B. diminuta* and diisopropylfluorophosphatase (DFPase) gene from squid *L. vulgaris* were used from previous studies [12,14]. The PTE gene encodes a 35.8 kDa protein (without tags, etc.), and the DFPase gene encodes a 34.9 kDa protein (without tags, etc.). The plasmids pET22b-OmpA-ST (truncated OmpA with the ST sequence appended to the N-terminus) and pACYC184 AraC PTE-SC were used as described earlier [12]. The DFPase-SC construct was generated using pACYC184 AraC PTE-SC as a template and replacing the PTE gene with the DFPase gene sequence using in vivo assembly [20]. In addition to DFPase-SC, PTE and DFPase were cloned into eight plasmids each: pET28b-Lpp’, pET28b-L3, pET28b-L4, pET28b-L34, pET22b-SlyB, pET22b-SLP, pET22b-BtuF, and pET22b-MPB using the same in vivo assembly method [20]. Sequences are provided in Table S1. The Lpp’ included a leader sequence of MKATKLVLGAVILGSTLLAG. In addition, mCherry was cloned into pET28b-Lpp’, pET28b-L3, pET28b-L4, and pET28b-L34. The sequences of all the constructs were verified by sequencing using Eurofins Genomics (Louisville, KY, USA).

### 2.3. NanoSight

The OMV size, volume, surface area, and count distributions were obtained on a NanoSight LM10 system (Malvern Instruments Ltd., Worcestershire, UK) using Nanoparticle Tracking and Analysis (NTA) 3.2 software. All samples were diluted 1:1000 in PBS or CHES with camera shutter and gain optimized for data collection. Three 90 s videos were recorded, and frame sequences were analyzed under auto particle detection and tracking parameters, including detection threshold, pixel blur, minimum track length, and minimum expected particle size. All samples were run at room temperature (RT).

### 2.4. mCherry Fluorescence

Fluorescence measurements for mCherry constructs were obtained on a BioTek Synergy Neo2 (Winooski, VT, USA). mCherry containing OMVs were read with excitation 587/5 nm and emission 610/5 nm. Each OMV preparation was normalized by concentration (particles/mL) as determined by NanoSight prior to measurement.

### 2.5. PTE and DFPase Activity Assay

All reagents were purchased from Sigma-Aldrich (St. Louis, MO, USA) unless otherwise noted. PTE assays were conducted in 50 mM CHES buffer (pH 8.5) at 25 °C in 384-well plates on a BioTek Synergy Neo2 (Winooski, VT, USA), with a final reaction volume of 20 µL. All samples were tested in biological replicates ≥8, in triplicate technical wells. OMV concentration was normalized by NanoSight (Malvern Instruments Ltd., Worcestershire, UK) such that each OMV-containing well contained the same number of OMVs. Paraoxon (1:1000 dilution from a neat concentration of ≈4.629 M) hydrolysis to p-nitrophenol was monitored using absorbance at 405 nm. Initial velocities were determined by the slope of the first 20 min of reaction with the paraoxon substrate. Log_2_ transformation was used in plots for easier visual analysis. DFPase activity assays were carried out identically to the PTE assays, but in 25 mM HEPES buffer (pH 7.0) with 2.5 mM CaCl_2_ instead of CHES at 30 °C.

### 2.6. SDS-PAGE and Western Blot Analysis

OMV samples were run on a gradient (4−15%) SDS-PAGE gel with a Tris-Glycine running buffer under reducing conditions at 120 V for 60 min and transferred to a nitrocellulose membrane at 15 V for 15 min in 10% methanol transfer buffer. The membrane was stained using Ponceau S, blocked in 3% skimmed milk in PBS-T (PBS with 0.1% Tween-20), and probed with 1:5000 dilution of mouse anti His-tag or anti-mCherry antibody overnight at 4 °C and a 1:5000 dilution of alkaline-phosphatase-conjugated anti-mouse secondary antibody at room temperature for 1 h. A chromogenic alkaline phosphatase NBT (nitro-blue tetrazolium chloride) and BCIP (5-bromo-4-chloro-3′-indolyphosphate p-toluidine salt) substrate solution was used to detect DFPase and mCherry. SDS-PAGE gels were stained with GelCode Blue for 30 min and destained for 4 h. All gel and blot images were taken on a Bio-Rad ChemiDoc imager (Hercules, CA, USA).

## 3. Results

### 3.1. PTE and DPTase OMV Packaging Strategy Design Rationale

In order to evaluate various different anchor/directing strategies and to investigate different linkers for packaging two organophosphate hydrolases, PTE and DFPase, into OMVs, we constructed a variety of different fusions (Figure 1 and detailed in Table 1). We were particularly interested in Lpp’, a truncated peptide variant (nine amino acids, CSSNAKIDQ) of Lpp, which is an outer membrane lipoprotein that has an N-terminal cysteine modified by a lipid. It has been previously used for anchoring proteins to the inner leaflet of the outer membrane via fusion to Lpp’ and for displaying proteins to the outer surface of the cell through fusion to a variant of OmpA [18]. We chose this anchor to further investigate the effect of linker characteristics between the anchor and the cargo enzyme. The first construct was Lpp’ directly fused to the N-terminus of the cargo enzyme with a short linker (12 amino acids, (GGGS)_3_). Then, sequences in addition to this short linker were added. The linker L3 (13 amino acids, ATGPASGPTSAGP) was designed to have glycine and proline somewhat uniformly spaced throughout the peptide to prevent any type of structure from forming, with small amino acids alanine and serine/threonine mixed in, which largely follows the repeated pattern of (A[T/S]GP) and is fused to Lpp’. The linker L4 (18 amino acids, PASPAPPAGPAPPAPTAP) is another fusion with Lpp’ derived from a rigid Pro-rich linker (PAPAP)N previously described by Zhao et al. [21,22]. Maintaining the proline/alanine-rich and semi-rigid sequence, it was adapted to the pattern (P-A-S/G/P/T) to simplify cloning using an overlap-PCR method. Finally, the linker L34 was constructed by combining L3 and L4 to create a longer-length 31 amino acid peptide. The relative lengths of these Lpp’-based linkers range from approximately 45 to 165 Å, assuming linearity. Each of these constructs was checked for a lack of predicted structure using protparam in ExPASy [23] prior to use. A C-terminus 6× His tag was appended in these fusion constructs to enable detection by immunoblot.

To compare different membrane anchors, in addition to Lpp’, we investigated OmpA, SlyB, and SLP. OmpA is a highly expressed protein found in OMVs and has been previously used by our group and others; in particular, we used a C-terminus truncated version and the SpyTag/SpyCatcher system we have shown previously to work well, and this acted as a type of positive benchmark for us [12]. To incorporate a different anchor likely to display the cargo enzyme on the outer surface of the OMV, we investigated SLP, an *E. coli* outer membrane lipoprotein associated with the outer membrane fraction of cells [24]. We also chose to test SlyB, a small outer membrane lipoprotein conserved in Gram-negative bacteria, which has previously been utilized as an anchor for packaging nanoluciferase into the lumen of OMVs [17].

As a different category of approach, periplasmic proteins were used, herein termed “directors”. The first was maltose-binding protein (MBP), which has been widely utilized as a fusion partner for improving the solubility of proteins [25] and directs proteins to the periplasmic space. The second was the *E. coli* vitamin-B12-binding protein (BtuF), a periplasmic binding protein for the vitamin B12 transporter BtuCD [26], which we hypothesized would direct fused proteins to the periplasm as it binds vitamin B12 and deliver it to partner proteins in the periplasmic space. Finally, as in our previous studies, we made use of the SpyCatcher-SpyTag (SC-ST) [11] system to drive the enzymes into the OMVs, but as an additional control fusion, we chose to test PTE/DFPase-SpyCatcher (SC) alone (without co-transforming with OmpA-ST), as at least one group has reported extensive non-specific binding of SC in isolation [27].

**Table 1:** Anchors/directors and linkers used in this study.

Name	Description/Design	Uniprot ID of Parent Protein	Membrane-Associated?	Reference
Lpp’	Trunicated, 9 amino acid peptide derived from Lpp outer membrane lipoprotein from *E. coli*; contains modified N-terminal Cys post-cleavage	P69776	Yes	[18]
L3	A short Gly-Pro-rich, semi-rigid linker appended to Lpp’	N/A	Yes	This work
L4	(P-A-[S/G/P/T])^N linker, based on the rigid proline-rich linker (PAPAP)^N; appends 50% protein linker to Lpp’	N/A	Yes	[21,22]
L34	L4 appended to L3 (Lpp’-L3-L4)	N/A	Yes	[14]
OpmA-ST-SC	The two-part SpyTag-SpyCatcher system, with OmpA-fused SpyTag and PTE or DFPase fused to SpyCatcher	P0A910	No	[11]
SC	PTE or DFPase fused to SpyCatcher; only the SpyCatcher protein component of the SpyTag-SpyCatcher system	N/A	No	[11,27]
BtuF	*E. coli* protein; periplasmic binding protein for the vitamin B12 transporter BtuCD	P37028	No	[26]
MBP	Maltose-binding protein	P0AEX9	No	[25]
SLP	Outer membrane lipoprotein; SLP may help to stabilize the outer membrane in stationary phase	P37194	Yes	[24]
SlyB	Small outer membrane lipoprotein conserved in Gram-negative bacteria	P0A905	Yes	[17]

**Figure 2:**
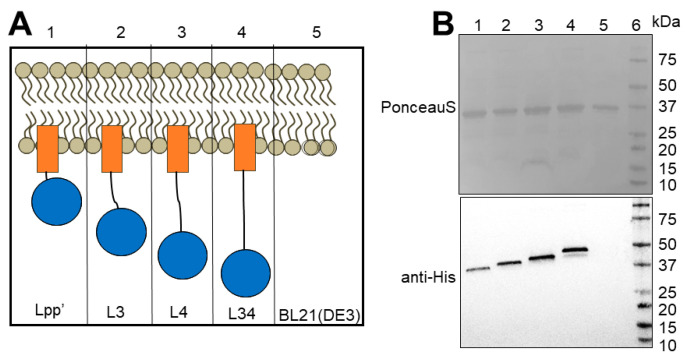
Western blot of purified OMVs from various constructs demonstrating the abundance of DFPase fused to Lpp’-based linkers. (**A**) Representation of the different DFPase fusion constructs. The orange box represents Lpp’ and the blue circle represents DFPase (**B**) Immunoblot lane 1: Lpp’-DFPase, lane 2: L3-DFPase, lane 3: L4-DFPase, lane 4: L34-DFPase, lane 5: BL21(DE3), lane 6: molecular weight marker. Upper panel shows the PonceauS-stained membrane, and the lower panel shows immunoblot with anti-6× His antibody.

The resulting constructs from the above strategies were transformed into *E. coli* BL21(DE3) and tested for packaging in OMVs (Figure 1, Table 1). As an initial test, we first wanted to examine whether the enzyme could be successfully packaged into OMVs. To perform this check, the OMVs containing DFPase fused to the Lpp’-based strategies were isolated and subjected to immunoblotting using anti-His antibody (Figure 2). The Western blot showed the presence of a band corresponding to DFPase in each case, and the apparent molecular weight increased as expected, with Lpp’, L3, L4, and L34 running in order of sequence length. Unlike DFPase, we were unable to quantitatively determine the levels of PTE in OMVs for the various linkers, possibly due to unexposed His-tags. Since we had previously demonstrated PTE packaging into OMVs using other strategies [12], and each construct displays a degree of activity against paraoxon (discussed below), this lack of initial packaging confirmation for PTE was determined as unnecessary.

### 3.2. Linker Type Affects OMV Production and Size

**Figure 3:**
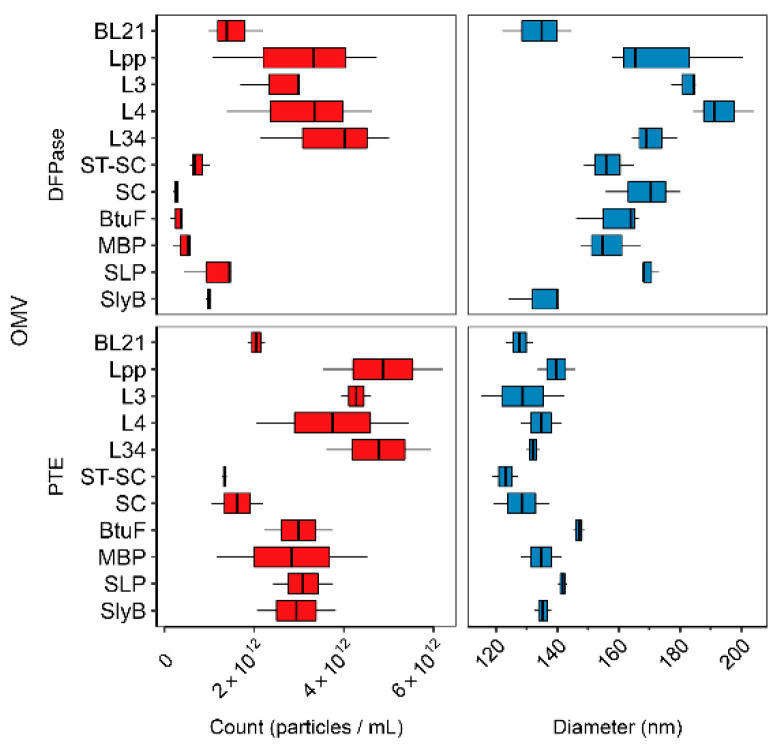
OMV size and count distributions. (**Left**) NanoSight count in particles/mL measured from each of the OMVs averaged over three 60 s sample reads of a 1:1000 diluted sample (in CHES pH 8.5). (**Right**) NanoSight size distribution from each of the OMVs.

Next, to characterize the enzyme-containing OMVs, we determined the distribution of sizes and performed quantification using NanoSight particle tracking. A summary of the results is presented in Figure 3. To measure the hyper- or hypo-vesiculation or impact on size resulting from each of the strategies, the OMVs were resuspended and diluted (1:1000) identically for accurate comparison. Here, each of the 10 linkers, Lpp’, L3, L4, L34, SC-ST, SC alone, BtuF, MBP, SLP, and SlyB, fused to either PTE or DFPase, or BL21(DE3) control, were examined for both OMV count (particles/mL) and diameter (nm). Interestingly, the OMVs expressing Lpp’-based fusions, as a group, produced significantly more OMVs than the others (Figure S1A, *p* < 0.05; Student’s *t*-test). This effect was more pronounced in the case of OMVs loaded with DFPase, as the difference was three- to fourfold greater particles/mL relative to SC-ST, SC alone, BtuF, MBP, SLP, and SlyB, whereas the reconstituted concentration of PTE-containing OMVs were higher overall, yielding a smaller difference between the Lpp’-based and non-Lpp’-based fusions.

In contrast, both PTE and DFPase OMVs containing SC only and the OmpA-ST-SC fusions yielded significantly lower counts compared to non-SC strategies that were uniformly higher in concentration (Figure 3 and Figure S1B, *p* < 0.05; Student’s *t*-test). The Lpp’-based fusions had high counts, although the same effect was not seen for SLP nor SlyB, which, along with the directors BtuF and MBP, demonstrated moderate OMV production (see Section 4 regarding hypervesiculation).

Next, we examined the relative impact the differing strategies had on OMV diameter. Unlike OMV concentration, very few noticeable trends in diameter were established when the packaging strategy was varied (Figure 2). However, certain notable trends existed, including the apparent increase in diameter that each DFPase OMV had (except SlyB) over the control BL21(DE3). Moreover, when compared to PTE-containing OMVs overall, the DFPase OMVs were found to be significantly larger (see Section 4).

### 3.3. Activity of OMV-Packaged Enzymes Was Influenced by Linkers

We next investigated the impact each of the packaging strategies had on the ability of the enzyme-loaded OMVs to hydrolyze the organophosphate paraoxon. Here, again, we tested the full suite of ten strategies, plus the enzyme-less BL21(DE3) control for both PTE and DFPase, where continuous monitoring of degradation of paraoxon to p-nitrophenol at 405 nm was used to determine relative impacts that each strategy had on activity. Since OMVs were normalized prior to testing, these experiments were not biased by the increased or suppressed vesiculation discussed in the previous section. Although paraoxon is hydrolyzed by both PTE and DFPase, the substrate is more preferred by PTE, which is reflected in the overall higher initial rates reported.

**Figure 4:**
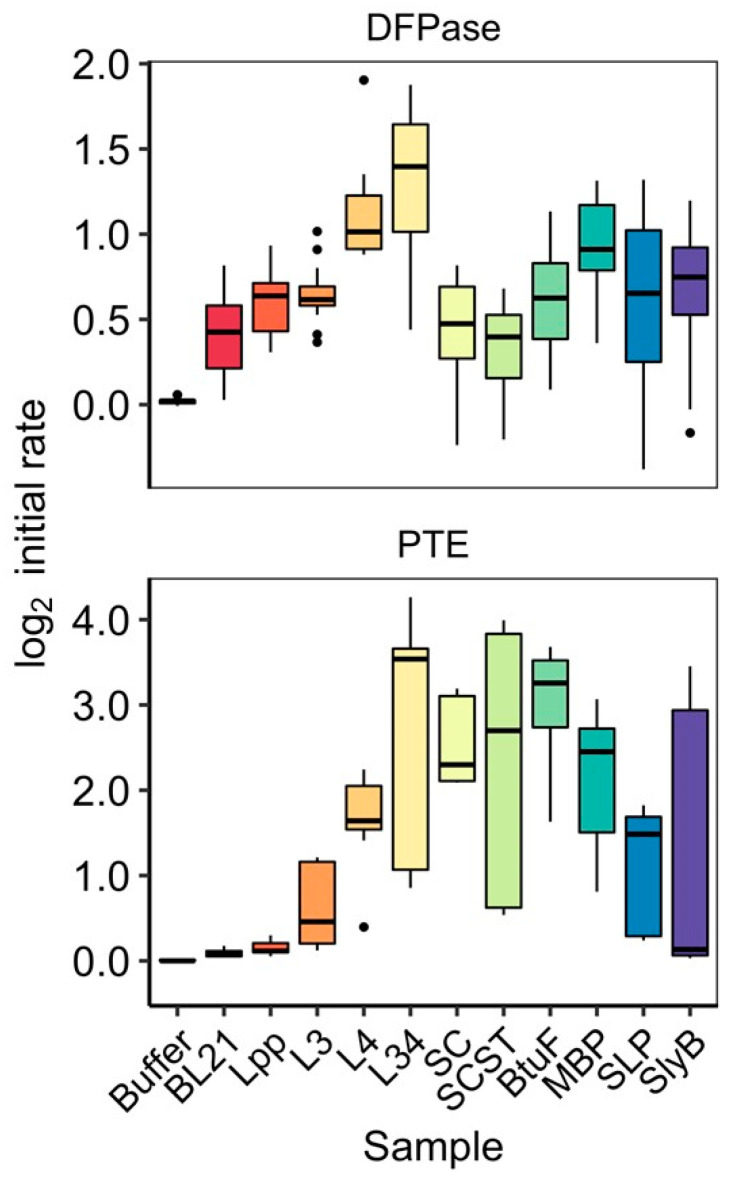
Enzyme activity of OMVs categorized by fusion and enzyme. Boxplot representations of the initial kinetic rates for the degradation of paraoxon by PTE (**top**) and DFPase (**bottom**) are shown (*n* ≥ 8), ordered by sample. Dots represent outliers. Initial rate was calculated from the first 20 min of the curve. Buffer did not contain OMVs, while BL21 was the BL21(DE3) OMV sample that did not contain PTE or DFPase.

The results, summarized in boxplots in Figure 4, demonstrate the large impact that individual strategies can have on the paraoxon-hydrolyzing activity (as measured by initial rate) of the OMVs, spanning a large range with the Lpp’ with L34 linker obtaining the highest median initial rate for both PTE and DFPase. Of note is the magnitude of the effect, whereby the L34-PTE OMV is approximately 11 times more active than its shorter parental linker, Lpp’; the activity disparity is similar for DFPase, with the median initial rate of L34-DFPase approximately three times higher than the lowest activity DFPase OMV, SC-ST. The clearest trend from these experiments is displayed by the series of Lpp’-based-linkers we generated, where a clear stepwise pattern results, with increasingly long fusions (in regards to sequence length) resulting in increasing activity for both PTE and DFPase. However, in the case of L3-DFPase, its median initial rate was determined to be slightly lower than that of Lpp’-DFPase, possibly the result of experimental variability. The high variability was more pronounced among certain groups of linkers, including the L34, SLP, and SlyB fusions for both enzymes, as well as ST-SC-PTE. Another interesting result was the contrast between the apparent activity of the SC-alone and SC-ST differences between PTE and DFPase. As can be seen in Figure 4, SC and SC-ST yielded among the highest activity OMVs for PTE, only behind in median L34-PTE and BtuF. In contrast, for DFPase, both SC and SC-ST OMVs resulted in the lowest activity, within the error of the BL21(DE3) control. Further, surprisingly, there was not much of a difference between SC-ST (containing the OmpA anchor) and SC alone, which both showed fairly low DPFase activity (packaging) for DFPase and fairly high activity for PTE. For DFPase, the periplasmic directors MPB worked moderately well and better than BtuF; for PTE, both ButF and MPB worked well, with BtuF performing better. For both DFPase and PTE, the outer membrane anchor SLP was fair. The anchor SlyB worked moderately well for DFPase but less well for PTE.

To further investigate the presence or absence of clear trends in the activity data, we studied whether any physiochemical characteristics of the fusions had clear correlations with the resulting activity of DFPase and PTE in OMVs. A summary of this analysis is found in Table 2, with correlations shown in Figure S2. Here, the log_2_ initial rates for each fusion-enzyme OMV sample was paired with calculated values for each fusion’s sequence (post-cleavage of the signal peptide, and not including the enzyme sequence). We looked at aliphatic index, boman index, net charge, hydrophobicity, instability index, pI, sequence length, and molecular weight (see Table 2). Interestingly, no characteristic was determined to be strongly correlated with the initial enzymatic rate of either enzyme. Of the results, the Boman index, a calculation of the potential for protein interaction proposed by Boman [28], was found to have the highest R^2^ of 0.42 for DFPase, suggesting a slight negative correlation between the Boman index of the examined strategies and the initial rate of DFPase (Figure S2). Less significant were the correlations of aliphatic index and instability index and DFPase activity, with R^2^ of 0.17 and 0.19, respectively. For PTE, only aliphatic index and hydrophobicity had values over 0.1, both with R^2^ of 0.1. Although there was a clear trend between linker length and activity for both PTE and DFPase among the Lpp’-based linker, as described above, when each of the other strategies were included in the length-activity correlation, no trend was seen. When the enzyme sequence was included in the calculations of each of the same characteristics, certain features continued to have intriguing levels of correlation, such as the Boman index (Table 2); however, the R^2^ values remained relatively low, and the length trend seen with Lpp’-based linkers remained the most interesting attribute in relation to increased enzymatic activity.

In order to better interrogate the impact linker length had on different cargos, we chose to use mCherry as a representative fluorescent protein. A set of mCherry fusions were created with Lpp’, Lpp’-L3, Lpp’-L4, and Lpp’-34, which were expressed and purified identically to the corresponding enzyme fusions. mCherry fluorescence was then measured for each of the resulting OMVs (Figure S3). To control for protein loading differences between the different mCherry OMVs, Coomassie staining and Western blots probing for mCherry were performed. Loading the same amount of total protein as measured by BCA assay, the Western blot showed approximately equal levels of mCherry expression and packaging into the purified vesicles (Figure S3A). The combined results of four experiments (*n* = 12) showed that the OMVs containing Lpp’-mCherry yielded the highest fluorescence, while the fluorescence from OMVs containing mCherry fused to longer linkers (L3, L4, and L34) was significantly lower, by ≈10-fold on average (*p* < 0.05; pairwise Student’s *t*-test) (Figure S3B). The increased fluorescence of the Lpp’-mCherry fusion, which was the most confined, with the lowest distance separation from the membrane inside the OMV, was consistent with previous studies that have shown increased confinement caused by small linker length resulting in increased fluorescence relative to yields from longer linkers [29].

## 4. Discussion and Conclusions

The ability to manipulate bacterial OMVs by targeting heterologous proteins either to the membrane or the lumen of the vesicles has extended their utility in therapeutics, bioremediation, bioimaging, and detection. In addition, OMVs are potent adjuvants and can stimulate both humoral and cell-mediated immunity and can therefore be employed in the generation of multi-valent vaccines. Although substantial research efforts have been dedicated to expanding their utility by packaging multiple enzymes in the OMVs, systemic study on the role of different anchoring strategies for packaging enzymes into OMVs is lacking. Some of the questions that remain unanswered are how different engineering strategies affect the packaging of proteins in the vesicles and how to identify the most appropriate strategies for efficiently delivering enzymes into the OMVs. While the strategy that sounds best and has been widely used for packaging enzymes in the OMVs is localizing the recombinant antigens on the vesicle surface, this mode can be challenging as it usually requires the construction of a chimera between the target protein and an endogenous outer membrane protein, and it is limited by the size of the passenger protein; moreover, the packaging efficiency reduces with increase in the number or size of the target enzyme. This also leads to another sea of queries: (1) whether to include a linker/spacer in between the target protein and the localization peptide, (2) if the linker is sufficiently long for efficient packaging and whether reducing or increasing the linker length will affect the packaging and activity of the enveloped enzymes, and (3) what sequence of the linker would be optimal for localization. Further, one could envision an alternate approach that would be to deliver a desired protein into the periplasm using a periplasm-localizing protein as a director.

To gain insight into these questions, we performed an evaluation of how different fusion systems, associated with membrane or freely localized in the lumen (anchors/directors), affect the vesiculation, packaging, and activity of enzymes. In addition, we performed a comprehensive test of linkers and demonstrated their effect on enzyme packaging, activity, count, and size distribution of vesicles using different recombinant protein systems. The most coherent finding was offered by the series of Lpp’-based-linkers we generated, wherein a clear stepwise pattern resulted, with increasingly long sequence fusions resulting in increasing activity for both PTE and DFPase. While we cannot with certainty use membrane proximity as the explanation, since it is possible the linker–enzyme fusion is folded over onto itself, particularly in the case of the flexible glycine-rich L3, previous studies have shown that proximity of enzymes and other proteins to the surfaces, such as membranes, is known to impact both enzymatic activity and fluorescence. This impact is largely attributed to both protein orientation and freedom of motion, with the longer linker reducing steric hindrance, as well as the differing microenvironments surrounding the proteins in these spaces [30,31].

Another interesting aspect noticed with Lpp’- and linker-based OMV localization is hypervesiculation. Interestingly, the standard lab strain of *E. coli* consistently produced more OMVs when transformed with Lpp’ fused to any length of linker, regardless of the enzyme system tested, with the length of the linker showing an additive effect on the amount of OMVs produced. Several hypervesiculating strains of bacteria, including *E. coli*, have been produced in the past by deletion of genes encoding for integral membrane proteins such as OmpA, TolB, and NlpI [32]. The property of the Lpp’-based strategies to promote hypervesiculation of *E. coli* indicates that this strategy has the potential to further enhance the OMV production when used in combination with the mutant strains previously tested by several groups. In line with our observations using Lpp’-based strategies, Irene et al. (2019) showed that lipidated proteins tend to be expressed at a higher level with respect to their nonlipidated counterparts during loading in OMVs [19]. Our results demonstrated an improvement in the enzyme activity upon incorporation of varied length linkers to the Lpp’ lipoprotein leader sequence, thereby expanding their utility in vaccine applications. This linker can be applied in the future to design strategies that could simultaneously lead to hypervesiculation and packaging of enzyme in native confirmation for activity or antigenicity.

While we noticed a similar trend with the linker studies with both PTE and DFPase, key differences were observed between the two enzymes when packaged using non-Lpp’-based strategies. One of the noticeable differences was seen with the SC-ST-based conjugation system used to drive the packaging of the enzymes. While PTE shows the highest activity using this approach, DFPase was minimally active. The low activity of DFPase might partly be explained by the relatively low expression level in OMVs when packaged using the SC-ST approach as compared to when packaged using the Lpp’-L34 approach [14]. Unfortunately, this comparison could not be made with PTE, as we were unable to visualize a band in immunoblot corresponding to PTE using Lpp’ linker-based strategies, perhaps due to unexposed His tags. However, we have previously shown that the expression of PTE is robust when packaged using the SC-ST approach [12]. Lastly, we also noticed efficient packaging and activity using PTE-SC alone (without SpyTag to drive packaging), indicating that PTE-SC associated with OMVs even in the absence of OmpA-ST and showed strong activity, implying that the sticky nature of PTE-SC also contributes to the overall activity. In conclusion, while the longer linker length approach can be successfully used for targeting a new enzyme in OMVs, the use of SC-ST-based strategies might have variable results with different enzyme systems and need to be tested on a case-by-case basis. Finally, regarding differences in PTE and DFPase packaging, we note that when compared to PTE-containing OMVs overall, the DFPase OMVs were found to be significantly larger, suggesting that the expression and packaging of the DFPase itself may result in OMVs of larger diameter, which could possibly be due to structural differences in-between the two enzymes or different expression levels [16].

Unlike recombinant enzymes, it is not feasible to perform structural characterization of the enzymes packaged in OMVs; however, the fact that both the enzymes were active with most of the packaging strategies employed strongly suggest that they preserved their native/active conformation. This is particularly important when packaging antigens for vaccine generation in order to elicit immune response mediated through conformational epitopes or packaging therapeutic and/or bioremediation enzymes where active conformation plays an important role. In summary, our data add to a growing number of strategies that can be employed to express heterologous proteins in bacterial OMVs and comprehensively dissects the role of different fusions systems that can be employed for functionalizing OMVs. The results of this study and future studies on linker and anchoring strategies are valuable for the potential use of OMVs as a platform for the delivery of therapeutic enzymes. Though the use of OMVs for therapeutics is still an emerging technology, they offer the capability of engineering systems to simultaneously produce both the therapeutic and targeting moiety in a single biological system [33].

## Figures and Tables

**Table 2:** Initial rate and linker characteristics.

Name	PTE Initial Rate (log_2_)	DFPase Intial Rate (log_2_)	pI	Boman Index	Mol. Weight	Charge	Hp ^1^	Length	Instability Index	Aliphatic Index
Lpp’	1.1	1.6	6.2	1.3	1739.7	−0.1	−0.06	21.0	49.9	23.3
L3	1.4	1.5	6.2	0.9	2791.9	−0.1	−0.5	34.0	37.8	23.2
L4	3.1	2.0	6.2	0.5	3285.5	−0.1	−0.5	39.0	108.3	27.9
L34	11.6	2.7	6.2	0.5	4337.6	−0.1	−0.4	52.0	81.1	26.7
BtuF	9.6	1.5	7.3	1.6	30,093.1	0.0	−0.4	274.0	39.6	89.7
MBP	5.5	1.9	4.8	1.4	43,378.0	−10.6	−0.4	396.0	20.8	81.4
SLP	2.8	1.6	6.5	1.5	22,299.9	−1.7	−0.4	201.0	34.5	81.9
SlyB	1.1	1.7	19.2	1.4	18,742.9	2.0	−0.1	185.0	28.2	87.8
SC	4.9	1.4	12.2	2.1	6540.3	3.5	−0.5	61.0	39.5	73.8

^1^ Hp = hydrophobicity.

## Data Availability

Data are contained within the article or Supplementary Material.

## Supplementary Materials

The following supporting information can be downloaded at https://www.mdpi.com/article/10.3390/bioengineering10050583/s1. Figure S1: Differences in count and diameter by linker categorization. Figure S2: Visual representation of correlations between linker physiochemical characteristics and enzymatic activity (log_2_ initial rate). Figure S3: Effect of Lpp’-based linkers on mCherry fluorescence. Table S1: Sequences of anchors/directors and linkers.
