# Supplementary material for: Different Strategies Affect Enzyme Packaging into Bacterial Outer Membrane Vesicles
Source: Bioengineering (Basel). 2023 May 11;10(5):583. doi: 10.3390/bioengineering10050583 (PMC10215610; doi:10.3390/bioengineering10050583)
Supplement: Supplementary file 1 [file bioengineering-10-00583-s001.zip › bioengineering-2374403-supplementary.pdf]

bioengineering

Supplementary Materials

Different Strategies Affect Enzyme Packaging into Bacterial Outer Membrane Vesicles

Scott N. Dean [1,†], Meghna Thakur [2,†], Joseph R. Spangler [1], Aaron D. Smith [1], Sean P. Garin [1,‡], Scott A. Walper [1,§] and Gregory A. Ellis [1,*]

1 Center for Bio/Molecular Science and Engineering, Code 6900, U.S. Naval Research Laboratory, Washington, DC 20375, USA
2 College of Science, George Mason University, Fairfax, VA 22030, USA; meghna.thakur.ctr.in@nrl.navy.mil
* Correspondence: gregory.ellis@nrl.navy.mil
† These authors contributed equally to this work.
‡ Current address: University of Maryland Medical Intelligent Imaging (UM2ii) Center, Department of Diagnostic Radiology and Nuclear Medicine, University of Maryland School of Medicine, Baltimore, MD 20201, USA.
§ Current address: US Office of Naval Research, 86 Blenheim Crescent, Ruislip, Middlesex HA4 7GB, UK.

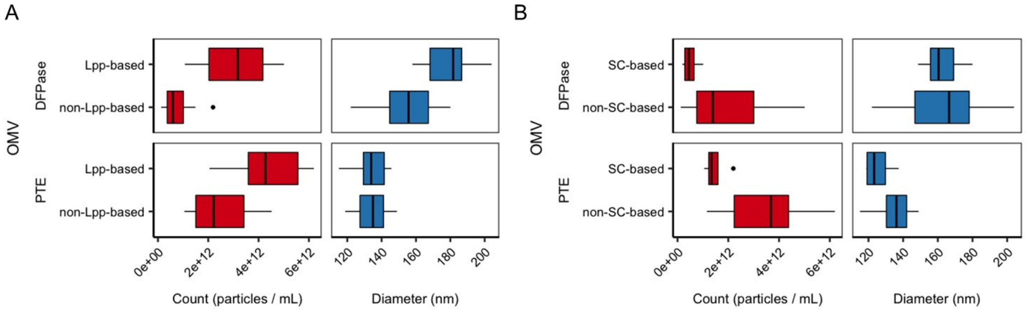

Figure S1. Differences in count and diameter by linker categorization. The count (particles/mL) and diameters (nm) are compared of (**A**) Lpp'-based versus non- Lpp'-based OMVs and (**B**) SC-based versus non-SC-based OMVs. Dots represent outliers.

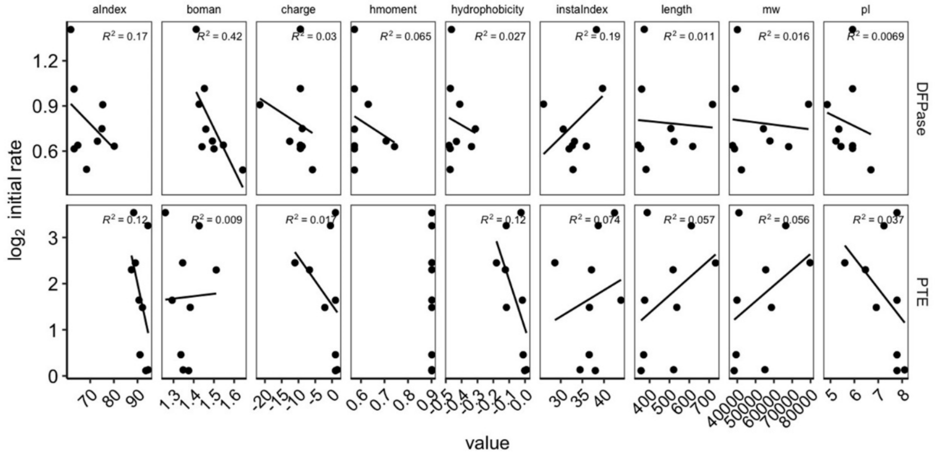

Figure S2. Visual representation of correlations between linker physiochemical characteristics and enzymatic activity (log2 initial rate). Initial rate taken from the linear portion of the first 20 minutes of the paraoxon degradation reaction. Sequence used for calculating characteristics does not include enzyme.

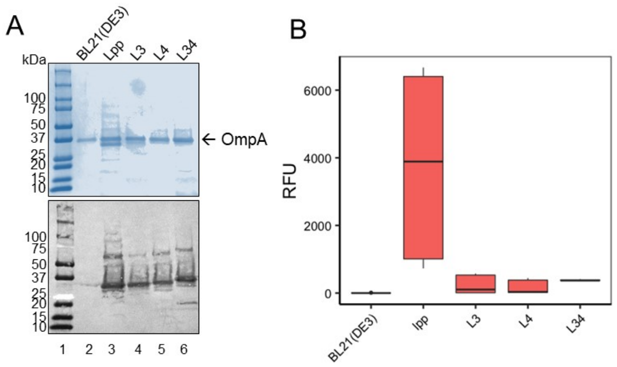

Figure S3. Effect of Lpp′-based linkers on mCherry fluorescence. (**A**) Coomassie gel of Lpp′-linker mCherry fusions (top) and corresponding immunoblot with anti-mCherry antibody (bottom) showing a major band at ~35 kDa in the total protein gel corresponding to the abundant OmpA, while 6× His-tagged-mCherry fusions are shown at increasing size corresponding to increasing linker length. Lane 1: Molecular weight marker, lane 2: BL21(DE3), lane 3: Lpp′-mCherry, lane 4: L3- mCherry, lane 5: L4- mCherry, lane 6: L34-mCherry. (**B**) Fluorescence intensity of OMVs from cultures producing Lpp′-based linkers fused to mCherry. Boxplot using data from four biological experiments in triplicate (n = 12).

Table S1. Sequences of anchors/directors and linkers.

**Name**	**Sequence**
Lpp′-PTE	TTTGTTTAACTTTAAGAAGGAGATATACCATGAAAGCGACCAAA<br>CTGGTGCTGGGCGCGGTGATTCTGGGCAGCACCCTGCTGGCGGG<br>C**TGCAGCAGCAACGCGAAAATTGATCAG**GGCGGTGGCAGCGG<br>CGGTGGCAGCGGCGGTGGGAGC*CGCATTAACACCGTGCGCGGCCC*<br>*GATTACCATTAGCGAAGCGGGCTTTACCCTGACCCATGAACATATTT*<br>*GCGGCAGCAGCGCGGGCTTTCTGCGCGCGTGGCCGGAATTTTTTGGC*<br>*AGCCGCAAAGCGCTGGCGGAAAAAGCGGTGCGCGGCCTGCGCCGCG*<br>*CGCGCGCGGCGGGCGTGCGCACCATTGTGGATGTGAGCACCTTTGAT*<br>*ATTGGCCGCGATGTGAGCCTGCTGGCGGAAGTGAGCCGCGCGGCGG*<br>*ATGTGCATATTGTGGCGGCGACAGGCCTGTGGTTTGATCCGCCGCTG*<br>*AGCATGCGCCTGCGCAGCGTGGAAGAACTGACCCAGTTTTTTCTGCG*<br>*CGAAATTCAGTATGGCATTGAAGATACCGGCATTCGCGCGGGCATTA*<br>*TTAAAGTGGCGACCACCGGCAAAGCGACCCCGTTTCAGGAACTGGT*<br>*GCTGAAAGCGGCGGCGCGCGCGAGCCTGGCGACCGGCGTGCCGGTG*<br>*ACCACCCATACCGCGGCGAGCCAGCGCGATGGCGAACAGCAGGCGG*<br>*CGATTTTTGAAAGATGATCTGAGCTATCTGACCGCGCTGGCGGCGCG*<br>*CGGCTATCTGATTGGCCCGAAGGCCTGAGCCCGAGCCGCGTGTGCAT*<br>*TGGCCATAGCGATGATACCGTGGATCATATTCCGCATAGCGCGATTG*<br>*GCCTGGAAGATAACGCGAGCGCGAGCGCGCTGCTGGGCATTCGCAG*<br>*CTGGCAGACCCGCGCGCTGCTGATTAAAGCGCTGATTGATCAGGGCT*<br>*ATATGAAACAGATTCTGGTGAGCAACGATTGGCTGTTTGGCTTTAGC*<br>*AGCTATGTGACCAACATTATGGATGTGATGGATCGCGTGAACCCGG*<br>*ATGGCATGGCGTTTATTCCGCTGCGCGTGATTCCGTTTCTGCGCGAA*<br>*AAAGGCGTGCCGCAGGAAACCCTGGCGGGCATTACCGTGACCAACC*<br>*CGGCGCGCTTTCTGAGCCCGACCCTGCGCGCGAGC* ***CACCACCACCA***<br>***CCACCAC***TGA
L3-PTE	TTTGTTTAACTTTAAGAAGGAGATATACCATGAAAGCGACCAAA<br>CTGGTGCTGGGCGCGGTGATTCTGGGCAGCACCCTGCTGGCGGG

C**TGCAGCAGCAACGCGAAAATTGATCA**GGGCGGTGGCAGCGG
CGGTGGCAGCGGCGGTGGGAGCGCCACCGGTCCGGCAAGCGGT
CCGACCAGCGCAGGTCCG*CGCATTAACACCGTGCGCGGCCCGATT*
ACCATTAGCGAAGCGGGCTTTACCCTGACCCATGAACATATTTGCGG
CAGCAGCGCGGGCTTTCTGCGCGCGTGGCCGGAATTTTTTGGCAGCC
GCAAAGCGCTGGCGGAAAAAGCGGTGCGCGGCCTGCGCCGCGCGCG
CGCGGCGGGCGTGCGCACCATTGTGGATGTGAGCACCTTTGATATTG
GCCGCGATGTGAGCCTGCTGGCGGAAGTGAGCCGCGCGGCGGATGT
GCATATTGTGGCGGCGACAGGCCTGTGGTTTGATCCGCCGCTGAGCA
TGCGCCTGCGCAGCGTGGAAGAACTGACCCAGTTTTTTCTGCGCGAA
ATTCAGTATGGCATTGAAGATACCGGCATTCGCGCGGGCATTATTAA
AGTGGCGACCACCGGCAAAGCGACCCCGTTTCAGGAACTGGTGCTG
AAAGCGGCGGCGCGCGCGAGCCTGGCGACCGGCGTGCCGGTGACCA
CCCATACCGCGGCGAGCCAGCGCGATGGCGAACAGCAGGCGGCGAT
TTTTGAAAGCGAAGGCCTGAGCCCGAGCCGCGTGTGCATTGGCCATA
GCGATGATACCGATGATCTGAGCTATCTGACCGCGCTGGCGGCGCGC
GGCTATCTGATTGGCCTGGATCATATTCCGCATAGCGCGATTGGCCT
GGAAGATAACGCGAGCGCGAGCGCGCTGCTGGGCATTCGCAGCTGG
CAGACCCGCGCGCTGCTGATTAAAGCGCTGATTGATCAGGGCTATAT
GAAACAGATTCTGGTGAGCAACGATTGGCTGTTTGGCTTTAGCAGCT
ATGTGACCAACATTATGGATGTGATGGATCGCGTGAACCCGGATGG
CATGGCGTTTATTCCGCTGCGCGTGATTCCGTTTCTGCGCGAAAAAG
GCGTGCCGCAGGAAACCCTGGCGGGCATTACCGTGACCAACCCGGC
*GCGCTTTCTGAGCCCGACCCTGCGCGCGAGC****CACCACCACCACCAC***
CACTGA

L4-PTE

TTTGTTTAACTTTAAGAAGGAGATATACCATGAAAGCGACCAAA
CTGGTGCTGGGCGCGGTGATTCTGGGCAGCACCCTGCTGGCGGG
C**TGCAGCAGCAACGCGAAAATTGATCA**GGGCGGTGGCAGCGG
CGGTGGCAGCGGCGGTGGGAGCCCGGCATCTCCGGCCCCGCCGG
CTGGTCCAGCACCGCCGGCCCCAACCGCACCG*CGCATTAACACCG*
TGCGCGGCCCGATTACCATTAGCGAAGCGGGCTTTACCCTGACCCAT
GAACATATTTGCGGCAGCAGCGCGGGCTTTCTGCGCGCGTGGCCGG
AATTTTTTGGCAGCCGCAAAGCGCTGGCGGAAAAAGCGGTGCGCGG
CCTGCGCCGCGCGCGCGCGGCGGGCGTGCGCACCATTGTGGATGTG
AGCACCTTTGATATTGGCCGCGATGTGAGCCTGCTGGCGGAAGTGAG
CCGCGCGGCGGATGTGCATATTGTGGCGGCGACAGGCCTGTGGTTTG
ATCCGCCGCTGAGCATGCGCCTGCGCAGCGTGGAAGAACTGACCCA
GTTTTTTCTGCGCGAAATTCAGTATGGCATTGAAGATACCGGCATTC
GCGCGGGCATTATTAAAGTGGCGACCACCGGCAAAGCGACCCCGTT
TCAGGAACTGGTGCTGAAAGCGGCGGCGCGCGCGAGCCTGGCGACC
GGCGTGCCGGTGACCACCCATACCGCGGCGAGCCAGCGCGATGGCG
AACAGCAGGCGGCGATTTTTGAAAGCGAAGGCCTGAGCCCGAGCCG
CGTGTGCATTGGCCATAGCGATGATACCGATGATCTGAGCTATCTGA
CCGCGCTGGCGGCGCGCGGCTATCTGATTGGCCTGGATCATATTCCG
CATAGCGCGATTGGCCTGGAAGATAACGCGAGCGCGAGCGCGCTGC
TGGGCATTCGCAGCTGGCAGACCCGCGCGCTGCTGATTAAAGCGCTG
ATTGATCAGGGCTATATGAAACAGATTCTGGTGAGCAACGATTGGCT
GTTTGGCTTTAGCAGCTATGTGACCAACATTATGGATGTGATGGATC
GCGTGAACCCGGATGGCATGGCGTTTATTCCGCTGCGCGTGATTCCG
TTTCTGCGCGAAAAAGGCGTGCCGCAGGAAACCCTGGCGGGCATTA

	CCGTGACCAACCCGGCGCGCTTTCTGAGCCCGACCCTGCGCGCGAGC<br>***CACCACCACCACCACCAC***TGA
L34-PTE	TTTGTTTAACTTTAAGAAGGAGATATACCATGAAAGCGACCAAA<br>CTGGTGCTGGGCGCGGTGATTCTGGGCAGCACCCTGCTGGCGGG<br>C**TGCAGCAGCAACGCGAAAATTGATCA**GGGCGGTGGCAGCGG<br>CGGTGGCAGCGGCGGTGGGAGCGCCACCGGTCCGGCAAGCGGT<br>CCGACCAGCGCAGGTCCGCCGGCATCTCCGGCCCCGCCGGCTGG<br>TCCAGCACCGCCGGCCCCAACCGCACCG*CGCATTAACACCGTGCG*<br>*CGGCCCGATTACCATTAGCGAAGCGGGCTTTACCCTGACCCATGAAC*<br>*ATATTTGCGGCAGCAGCGCGGGCTTTCTGCGCGCGTGGCCGGAATTT*<br>*TTTGGCAGCCGCAAAGCGCTGGCGGAAAAAGCGGTGCGCGGCCTGC*<br>*GCCGCGCGCGCGCGGCGGGCGTGCGCACCATTGTGGATGTGAGCAC*<br>*CTTTGATATTGGCCGCGATGTGAGCCTGCTGGCGGAAGTGAGCCGCG*<br>*CGGCGGATGTGCATATTGTGGCGGCGACAGGCCTGTGGTTTGATCCG*<br>*CCGCTGAGCATGCGCCTGCGCAGCGTGGAAGAACTGACCCAGTTTTT*<br>*TCTGCGCGAAATTCAGTATGGCATTGAAGATACCGGCATTCGCGCGG*<br>*GCATTATTAAAGTGGCGACCACCGGCAAAGCGACCCCGTTTCAGGA*<br>*ACTGGTGCTGAAAGCGGCGGCGCGCGCGAGCCTGGCGACCGGCGTG*<br>*CCGGTGACCACCCATACCGCGGCGAGCCAGCGCGATGGCGAACAGC*<br>*AGGCGGCGATTTTTGAAAGCGAAGGCCTGAGCCCGAGCCGCGTGTG*<br>*CATTGGCCATAGCGATGATACCGATGATCTGAGCTATCTGACCGCGC*<br>*TGGCGGCGCGCGGCTATCTGATTGGCCTGGATCATATTCCGCATAGC*<br>*GCGATTGGCCTGGAAGATAACGCGAGCGCGAGCGCGCTGCTGGGCA*<br>*TTCGCAGCTGGCAGACCCGCGCGCTGCTGATTAAAGCGCTGATTGAT*<br>*CAGGGCTATATGAAACAGATTCTGGTGAGCAACGATTGGCTGTTTGG*<br>*CTTTAGCAGCTATGTGACCAACATTATGGATGTGATGGATCGCGTGA*<br>*ACCCGGATGGCATGGCGTTTATTCCGCTGCGCGTGATTCCGTTTCTG*<br>*CGCGAAAAAGGCGTGCCGCAGGAAACCCTGGCGGGCATTACCGTGA*<br>*CCAACCCGGCGCGCTTTCTGAGCCCGACCCTGCGCGCGAGC****CACCAC***<br>***CACCACCACCAC***TGA
BtuF-PTE	atgGCTAAGTCACTGTTCAGGGCGCTGGTCGCCCTGTCTTTTCTTGC<br>GCCACTGTGGCTCAACGCCGCGCCGCGCGTCATCACGCTTTCTCC<br>CGCCAACACTGAACTTGCCTTTGCCGCCGGGATCACGCCGGTTG<br>GGGTCAGCAGCTATTCCGACTATCCTCCACAAGCGCAAAAGATT<br>GAGCAGGTTTCCACCTGGCAGGGGATGAATCTGGAACGCATTGT<br>CGCGCTGAAACCCGATCTGGTGATTGCCTGGCGTGGAGGTAATG<br>CCGAGCGGCAGGTTGACCAGCTGGCTTCGCTGGGAATAAAAGTG<br>ATGTGGGTCGATGCGACAAGCATTGAACAAATTGCCAATGCGTT<br>ACGTCAACTGGCCCCCTGGAGTCCGCAACCAGACAAGGCCGAA<br>CAAGCCGCGCAATCCCTGCTGGATCAGTACGCGCAATTGAAAGC<br>GCAATATGCTGATAAACCTAAAAAACGTGTTTTTCTGCAATTCGG<br>CATTAATCCGCCATTTACCAGTGGAAAAGAGTCGATTCAGAACC<br>AGGTACTCGAAGTTTGTGGCGGAGAAAACATCTTTAAAGACAGC<br>CGGGTTCCCTGGCCGCAAGTTAGCCGCGAACAGGTGTTAGCACG<br>CTCGCCACAGGCGATTGTCATTACAGGCGGACCGGACCAAATTC<br>CTAAAATCAAACAATACTGGGGTGAACAGCTCAAAATTCCCGTT<br>ATTCCTCTCACGAGTGACTGGTTTGAACGTGCAAGCCCACGTATT<br>ATCCTCGCTGCACAACAGCTCTGTAATGCGCTTTCACAGGTAGAT<br>AATTCGAGCTCGAACAACAACAACAATAACAATAACAACAACC

TCGGGATCGAGGGAAGGATTTCACATATGAGCATTGGCACCGGC
GAT*CGCATTAACACCGTGCGCGGCCCGATTACCATTAGCGAAGCGG*
GCTTTACCCTGACCCATGAACATATTTGCGGCAGCAGCGCGGGCTTT
CTGCGCGCGTGGCCGGAATTTTTTGGCAGCCGCAAAGCGCTGGCGG
AAAAAGCGGTGCGCGGCCTGCGCCGCGCGCGCGCGGCGGGCGTGCG
CACCATTGTGGATGTGAGCACCTTTGATATTGGCCGCGATGTGAGCC
TGCTGGCGGAAGTGAGCCGCGCGGCGGATGTGCATATTGTGGCGGC
GACAGGCCTGTGGTTTGATCCGCCGCTGAGCATGCGCCTGCGCAGCG
TGGAAGAACTGACCCAGTTTTTTCTGCGCGAAATTCAGTATGGCATT
GAAGATACCGGCATTCGCGCGGGCATTATTAAAGTGGCGACCACCG
GCAAAGCGACCCCGTTTCAGGAACTGGTGCTGAAAGCGGCGGCGCG
CGCGAGCCTGGCGACCGGCGTGCCGGTGACCACCCATACCGCGGCG
AGCCAGCGCGATGGCGAACAGCAGGCGGCGATTTTTGAAAGCGAAG
GCCTGAGCCCGAGCCGCGTGTGCATTGGCCATAGCGATGATACCGAT
GATCTGAGCTATCTGACCGCGCTGGCGGCGCGCGGCTATCTGATTGG
CCTGGATCATATTCCGCATAGCGCGATTGGCCTGGAAGATAACGCGA
GCGCGAGCGCGCTGCTGGGCATTCGCAGCTGGCAGACCCGCGCGCT
GCTGATTAAAGCGCTGATTGATCAGGGCTATATGAAACAGATTCTGG
TGAGCAACGATTGGCTGTTTGGCTTTAGCAGCTATGTGACCAACATT
ATGGATGTGATGGATCGCGTGAACCCGGATGGCATGGCGTTTATTCC
GCTGCGCGTGATTCCGTTTCTGCGCGAAAAAGGCGTGCCGCAGGAA
ACCCTGGCGGGCATTACCGTGACCAACCCGGCGCGCTTTCTGAGCCC
*GACCCTGCGCGCGAGC*GAATTCCCTGCAGGTAATTAA

MBP-PTE

ATGAAAATAAAAACAGGTGCACGCATCCTCGCATTATCCGCATT
AACGACGATGATGTTTTCCGCCTCGGCTCTCGCCAAAATCGAAG
AAGGTAAACTGGTAATCTGGATTAACGGCGATAAAGGCTATAAC
GGTCTCGCTGAAGTCGGTAAGAAATTCGAGAAAGATACCGGAAT
TAAAGTCACCGTTGAGCATCCGGATAAACTGGAAGAGAAATTCC
CACAGGTTGCGGCAACTGGCGATGGCCCTGACATTATCTTCTGGG
CACACGACCGCTTTGGTGGCTACGCTCAATCTGGCCTGTTGGCTG
AAATCACCCCGGACAAAGCGTTCCAGGACAAGCTGTATCCGTTT
ACCTGGGATGCCGTACGTTACAACGGCAAGCTGATTGCTTACCC
GATCGCTGTTGAAGCGTTATCGCTGATTTATAACAAAGATCTGCT
GCCGAACCCGCCAAAAACCTGGGAAGAGATCCCGGCGCTGGAT
AAAGAACTGAAAGCGAAAGGTAAGAGCGCGCTGATGTTCAACC
TGCAAGAACCGTACTTCACCTGGCCGCTGATTGCTGCTGACGGG
GGTTATGCGTTCAAGTATGAAAACGGCAAGTACGACATTAAAGA
CGTGGGCGTGGATAACGCTGGCGCGAAAGCGGGTCTGACCTTCC
TGGTTGACCTGATTAAAAACAAACACATGAATGCAGACACCGAT
TACTCCATCGCAGAAGCTGCCTTTAATAAAGGCGAAACAGCGAT
GACCATCAACGGCCCGTGGGCATGGTCCAACATCGACACCAGCA
AAGTGAATTATGGTGTAACGGTACTGCCGACCTTCAAGGGTCAA
CCATCCAAACCGTTCGTTGGCGTGCTGAGCGCAGGTATTAACGC
CGCCAGTCCGAACAAAGAGCTGGCAAAAGAGTTCCTCGAAAAC
TATCTGCTGACTGATGAAGGTCTGGAAGCGGTTAATAAAGACAA
ACCGCTGGGTGCCGTAGCGCTGAAGTCTTACGAGGAAGAGTTGG
TGAAAGATCCGCGTATTGCCGCCACTATGGAAAACGCCCAGAAA
GGTGAAATCATGCCGAACATCCCGCAGATGTCCGCTTTCTGGTAT
GCCGTGCGTACTGCGGTGATCAACGCCGCCAGCGGTCGTCAGAC
TGTCGATGAAGCCCTGAAAGACGCGCAGACTAATTCGAGCTCGA

ACAACAACAACAATAACAATAACAACAACCTCGGGATCGAGGG
AAGGATTTCACATATGAGCATTGGCACCGGCGAT*CGCATTAACAC*
CGTGCGCGGCCCGATTACCATTAGCGAAGCGGGCTTTACCCTGACCC
ATGAACATATTTGCGGCAGCAGCGCGGGCTTTCTGCGCGCGTGGCCG
GAATTTTTTGGCAGCCGCAAAGCGCTGGCGGAAAAAGCGGTGCGCG
GCCTGCGCCGCGCGCGCGCGGCGGGCGTGCGCACCATTGTGGATGT
GAGCACCTTTGATATTGGCCGCGATGTGAGCCTGCTGGCGGAAGTGA
GCCGCGCGGCGGATGTGCATATTGTGGCGGCGACAGGCCTGTGGTTT
GATCCGCCGCTGAGCATGCGCCTGCGCAGCGTGGAAGAACTGACCC
AGTTTTTTCTGCGCGAAATTCAGTATGGCATTGAAGATACCGGCATT
CGCGCGGGCATTATTAAAGTGGCGACCACCGGCAAAGCGACCCCGT
TTCAGGAACTGGTGCTGAAAGCGGCGGCGCGCGCGAGCCTGGCGAC
CGGCGTGCCGGTGACCACCCATACCGCGGCGAGCCAGCGCGATGGC
GAACAGCAGGCGGCGATTTTTGAAAGCGAAGGCCTGAGCCCGAGCC
GCGTGTGCATTGGCCATAGCGATGATACCGATGATCTGAGCTATCTG
ACCGCGCTGGCGGCGCGCGGCTATCTGATTGGCCTGGATCATATTCC
GCATAGCGCGATTGGCCTGGAAGATAACGCGAGCGCGAGCGCGCTG
CTGGGCATTCGCAGCTGGCAGACCCGCGCGCTGCTGATTAAAGCGCT
GATTGATCAGGGCTATATGAAACAGATTCTGGTGAGCAACGATTGG
CTGTTTGGCTTTAGCAGCTATGTGACCAACATTATGGATGTGATGGA
TCGCGTGAACCCGGATGGCATGGCGTTTATTCCGCTGCGCGTGATTC
CGTTTCTGCGCGAAAAAGGCGTGCCGCAGGAAACCCTGGCGGGCAT
TACCGTGACCAACCCGGCGCGCTTTCTGAGCCCGACCCTGCGCGCGA
*GC*GAATTCCCTGCAGGTAATTAA

SLP-PTE

ATGAACATGACAAAAGGTGCACTCATCCTCAGCCTTTCATTTTTG
CTTGCCGCATGTAGTTCAATTCCGCAAAATATCAAAGGCAATAA
CCAACCTGATATTCAAAAAAGTTTTGTTGCTGTTCATAACCAGCC
GGGGTTATATGTTGGTCAACAAGCGCGCTTTGGTGGGAAGGTTAT
CAACGTTATCAATGGCAAAACGGATACGTTGTTAGAAATCTCTGT
ATTACCGTTGGATAGCTATGCGAAGCCTGATATTGAAGCCAACT
ATCAGGGCCGACTGCTCGCCAGACAAAGCGGCTTCCTTGATCCA
GTGAACTATCGTAATCACTTTGTTACCATCCTCGGCACCATTCAG
GGTGAACAACCTGGCTTTATCAATAAAGTCCCGTATAACTTCCTG
GAAGTGAATATGCAGGGCATCCAGGTGTGGCATTTGAGAGAAGT
GGTTAATACCACCTATAACCTGTGGGATTACGGCTATGGTGCATT
CTGGCCGGAACCGGGCTGGGGTGCGCCTTACTACACCAATGCGG
TGAGTCAGGTAACACCTGAGCTGGTCAAAAATTCGAGCTCGAAC
AACAACAACAATAACAATAACAACAACCTCGGGATCGAGGGAA
GGATTTCACATATGAGCATTGGCACCGGCGAT*CGCATTAACACCG*
TGCGCGGCCCGATTACCATTAGCGAAGCGGGCTTTACCCTGACCCAT
GAACATATTTGCGGCAGCAGCGCGGGCTTTCTGCGCGCGTGGCCGG
AATTTTTTGGCAGCCGCAAAGCGCTGGCGGAAAAAGCGGTGCGCGG
CCTGCGCCGCGCGCGCGCGGCGGGCGTGCGCACCATTGTGGATGTG
AGCACCTTTGATATTGGCCGCGATGTGAGCCTGCTGGCGGAAGTGAG
CCGCGCGGCGGATGTGCATATTGTGGCGGCGACAGGCCTGTGGTTTG
ATCCGCCGCTGAGCATGCGCCTGCGCAGCGTGGAAGAACTGACCCA
GTTTTTTCTGCGCGAAATTCAGTATGGCATTGAAGATACCGGCATTC
GCGCGGGCATTATTAAAGTGGCGACCACCGGCAAAGCGACCCCGTT
TCAGGAACTGGTGCTGAAAGCGGCGGCGCGCGCGAGCCTGGCGACC
GGCGTGCCGGTGACCACCCATACCGCGGCGAGCCAGCGCGATGGCG

AACAGCAGGCGGCGATTTTTGAAAGCGAAGGCCTGAGCCCGAGCCG
CGTGTGCATTGGCCATAGCGATGATACCGATGATCTGAGCTATCTGA
CCGCGCTGGCGGCGCGCGGCTATCTGATTGGCCTGGATCATATTCCG
CATAGCGCGATTGGCCTGGAAGATAACGCGAGCGCGAGCGCGCTGC
TGGGCATTCGCAGCTGGCAGACCCGCGCGCTGCTGATTAAAGCGCTG
ATTGATCAGGGCTATATGAAACAGATTCTGGTGAGCAACGATTGGCT
GTTTGGCTTTAGCAGCTATGTGACCAACATTATGGATGTGATGGATC
GCGTGAACCCGGATGGCATGGCGTTTATTCCGCTGCGCGTGATTCCG
TTTCTGCGCGAAAAAGGCGTGCCGCAGGAAACCCTGGCGGGCATTA
CCGTGACCAACCCGGCGCGCTTTCTGAGCCCGACCCTGCGCGCGAGC
GAATTCCCTGCAGGTAATTAAAACACCTGAGCTGGTCAAA

SlyB-PTE

ATGATTAAACGCGTATTGGTTGTTTCAATGGTAGGTCTGTCTCTTG
TCGGTTGTGTTAATAACGACACCCTGTCAGGGGATGTTTATACCG
CTTCTGAAGCGAAACAAGTACAGAATGTCAGCTATGGCACCATC
GTTAACGTACGTCCGGTACAGATTCAGGGCGGTGATGATTCCAA
CGTTATCGGTGCAATTGGCGGTGCTGTTCTTGGTGGTTTCCTGGGG
AATACTGTTGGTGGCGGAACCGGGCGTTCTCTGGCTACTGCAGC
AGGCGCTGTTGCAGGTGGCGTAGCTGGTCAGGGCGTACAGAGTG
CAATGAACAAAACGCAGGGTGTCGAGCTGGAAATTCGTAAAGA
CGATGGTAATACCATCATGGTGGTACAGAAACAAGGCAACACTC
GTTTCTCTCCGGGCCAACGTGTCGTACTGGCCAGCAATGGCAGTC
AGGTGACCGTTTCTCCGCGCAATTCGAGCTCGAACAACAACAAC
AATAACAATAACAACAACCTCGGGATCGAGGGAAGGATTTCAC
ATATGAGCATTGGCACCGGCGAT*CGCATTAACACCGTGCGCGGCC*
CGATTACCATTAGCGAAGCGGGCTTTACCCTGACCCATGAACATATT
TGCGGCAGCAGCGCGGGCTTTCTGCGCGCGTGGCCGGAATTTTTGG
CAGCCGCAAAGCGCTGGCGGAAAAAGCGGTGCGCGGCCTGCGCCGC
GCGCGCGCGGCGGGCGTGCGCACCATTGTGGATGTGAGCACCTTTG
ATATTGGCCGCGATGTGAGCCTGCTGGCGGAAGTGAGCCGCGCGGC
GGATGTGCATATTGTGGCGGCGACAGGCCTGTGGTTTGATCCGCCGC
TGAGCATGCGCCTGCGCAGCGTGGAAGAACTGACCCAGTTTTTTCTG
CGCGAAATTCAGTATGGCATTGAAGATACCGGCATTCGCGCGGGCA
TTATTAAAGTGGCGACCACCGGCAAAGCGACCCCGTTTCAGGAACT
GGTGCTGAAAGCGGCGGCGCGCGCGAGCCTGGCGACCGGCGTGCCG
GTGACCACCCATACCGCGGCGAGCCAGCGCGATGGCGAACAGCAGG
CGGCGATTTTTGAAAGCGAAGGCCTGAGCCCGAGCCGCGTGTGCATT
GGCCATAGCGATGATACCGATGATCTGAGCTATCTGACCGCGCTGGC
GGCGCGCGGCTATCTGATTGGCCTGGATCATATTCCGCATAGCGCGA
TTGGCCTGGAAGATAACGCGAGCGCGAGCGCGCTGCTGGGCATTCG
CAGCTGGCAGACCCGCGCGCTGCTGATTAAAGCGCTGATTGATCAG
GGCTATATGAAACAGATTCTGGTGAGCAACGATTGGCTGTTTGGCTT
TAGCAGCTATGTGACCAACATTATGGATGTGATGGATCGCGTGAACC
CGGATGGCATGGCGTTTATTCCGCTGCGCGTGATTCCGTTTCTGCGC
GAAAAAGGCGTGCCGCAGGAAACCCTGGCGGGCATTACCGTGACCA
*ACCCGGCGCGCTTTCTGAGCCCGACCCTGCGCGCGAGC*GAATTCCC
TGCAGGTAATTAA

Lpp'-DFPase

TTTGTTTAACTTTAAGAAGGAGATATACCATGAAAGCGACCAAA
CTGGTGCTGGGCGCGGTGATTCTGGGCAGCACCCTGCTGGCGGG
C**TGCAGCAGCAACGCGAAAATTGATCA**GGGCGGTGGCAGCGG

	CGGTGGCAGCGGCGGTGGGAGC*ATGGGGGAAATCCCTGTCATTGA*<br>*GCCGCTGTTTACCAAAGTCACCGAAGATATCCCGGGTGCCGAGGGTC*<br>*CGGTGTTCGACAAAAACGGTGACTTTTACATCGTTGCGCCGGCAGTT*<br>*GAAGTGAATGGCAAGCCTGCTGGTGAGATTCTGCGTATTGACCTGAA*<br>*AACGGGCAAGAAAACGGTGATTTGTAAACCTGAAGTTAATGGTTAT*<br>*GGCGGCATCCCGGCCGGTTGCCAATGCGATCGTGATGCCAACCAACT*<br>*GTTCGTGGCAGACATGCGCCTGGGCCTGTTGGTCGTTCAGACCGACG*<br>*GCACCTTCGAAGAAATCGCGAAAAAAGACTCCGAGGGTCGTCGTAT*<br>*GCAGGGCTGCAATGACTGCGCGTTCGACTACGAGGGCAATCTGTGG*<br>*ATTACCGCGCCAGCGGGCGAAGTCGCTCCGGCGGATGCTACCGCGA*<br>*GCATGCAAGAAAAGTTCGGTAGCATTTATTGTTTCACGACTGATGGC*<br>*CAGATGATTCAAGTCGATACGGCGTTCCAATTTCCGAATGGTATTGC*<br>*GGTCCGTCACATGAACGATGGTCGCCCGTACCAGCTGATCGTTGCCG*<br>*AGATGCCGACTAAGAAATTGTGGTCGTACGACATCAAGGGTCCGGC*<br>*AAAGATTGAGAACAAAAAAGTGTGGGGTCACATTCCGGGTACGCAT*<br>*GAGGGTGGCGCGGATGGTATGGATTTTGACGAAGATAACAACTTGC*<br>*TGGTTGCAAATTGGGGTTCTAGCCACATCGAAGTCTTTGGTCCGGAT*<br>*GGTGGCCAGCCGAAGATGCGCATCCGTTGCCCGTTCGAGAAGCCGA*<br>*GCAATCTGCACTTCAAGCCGCAGACCAAGACGATTTTTGTGACCGAG*<br>*CATGAGAACAATGCAGTTTGGAAATTTGAGTGGCAACGTAACGGTA*<br>*AGAAACAGTATTGTGAAACCCTGAAGTTTGGTATCTTC****CACCACCAC***<br>***CACCACCAC***TGA
L3- DFPase	TTTGTTTAACTTTAAGAAGGAGATATACCATGAAAGCGACCAAA<br>CTGGTGCTGGGCGCGGTGATTCTGGGCAGCACCCTGCTGGCGGG<br>C**TGCAGCAGCAACGCGAAAATTGATCA**GGGCGGTGGCAGCGG<br>CGGTGGCAGCGGCGGTGGGAGCGCCACCGGTCCGGCAAGCGGT<br>CCGACCAGCGCAGGTCCG*ATGGGGGAAATCCCTGTCATTGAGCCG*<br>*CTGTTTACCAAAGTCACCGAAGATATCCCGGGTGCCGAGGGTCCGGT*<br>*GTTCGACAAAAACGGTGACTTTTACATCGTTGCGCCGGCAGTTGAAG*<br>*TGAATGGCAAGCCTGCTGGTGAGATTCTGCGTATTGACCTGAAAACG*<br>*GGCAAGAAAACGGTGATTTGTAAACCTGAAGTTAATGGTTATGGCG*<br>*GCATCCCGGCCGGTTGCCAATGCGATCGTGATGCCAACCAACTGTTC*<br>*GTGGCAGACATGCGCCTGGGCCTGTTGGTCGTTCAGACCGACGGCAC*<br>*CTTCGAAGAAATCGCGAAAAAAGACTCCGAGGGTCGTCGTATGCAG*<br>*GGCTGCAATGACTGCGCGTTCGACTACGAGGGCAATCTGTGGATTAC*<br>*CGCGCCAGCGGGCGAAGTCGCTCCGGCGGATGCTACCGCGAGCATG*<br>*CAAGAAAAGTTCGGTAGCATTTATTGTTTCACGACTGATGGCCAGAT*<br>*GATTCAAGTCGATACGGCGTTCCAATTTCCGAATGGTATTGCGGTCC*<br>*GTCACATGAACGATGGTCGCCCGTACCAGCTGATCGTTGCCGAGATG*<br>*CCGACTAAGAAATTGTGGTCGTACGACATCAAGGGTCCGGCAAAGA*<br>*TTGAGAACAAAAAAGTGTGGGGTCACATTCCGGGTACGCATGAGGG*<br>*TGGCGCGGATGGTATGGATTTTGACGAAGATAACAACTTGCTGGTTG*<br>*CAAATTGGGGTTCTAGCCACATCGAAGTCTTTGGTCCGGATGGTGGC*<br>*CAGCCGAAGATGCGCATCCGTTGCCCGTTCGAGAAGCCGAGCAATC*<br>*TGCACTTCAAGCCGCAGACCAAGACGATTTTTGTGACCGAGCATGAG*<br>*AACAATGCAGTTTGGAAATTTGAGTGGCAACGTAACGGTAAGAAAC*<br>*AGTATTGTGAAACCCTGAAGTTTGGTATCTTC****CACCACCACCACCAC***<br>***CAC***TGA

L4- DFPase	TTTGTTTAACTTTAAGAAGGAGATATACCATGAAAGCGACCAAA<br>CTGGTGCTGGGCGCGGTGATTCTGGGCAGCACCCTGCTGGCGGG<br>C**TGCAGCAGCAACGCGAAAATTGATCA**GGGCGGTGGCAGCGG<br>CGGTGGCAGCGGCGGTGGGAGCCCGGCATCTCCGGCCCCGCCGG<br>CTGGTCCAGCACCGCCGGCCCCAACCGCACCG*ATGGGGGAAATC*<br>*CCTGTCATTGAGCCGCTGTTTACCAAAGTCACCGAAGATATCCCGGG*<br>*TGCCGAGGGTCCGGTGTTCGACAAAAACGGTGACTTTTACATCGTTG*<br>*CGCCGGCAGTTGAAGTGAATGGCAAGCCTGCTGGTGAGATTCTGCGT*<br>*ATTGACCTGAAAACGGGCAAGAAAACGGTGATTTGTAAACCTGAAG*<br>*TTAATGGTTATGGCGGCATCCCGGCCGGTTGCCAATGCGATCGTGAT*<br>*GCCAACCAACTGTTCGTGGCAGACATGCGCCTGGGCCTGTTGGTCGT*<br>*TCAGACCGACGGCACCTTCGAAGAAATCGCGAAAAAAGACTCCGAG*<br>*GGTCGTCGTATGCAGGGCTGCAATGACTGCGCGTTCGACTACGAGG*<br>*GCAATCTGTGGATTACCGCGCCAGCGGGCGAAGTCGCTCCGGCGGA*<br>*TGCTACCGCGAGCATGCAAGAAAAGTTCGGTAGCATTTATTGTTTCA*<br>*CGACTGATGGCCAGATGATTCAAGTCGATACGGCGTTCCAATTTCCG*<br>*AATGGTATTGCGGTCCGTCACATGAACGATGGTCGCCCGTACCAGCT*<br>*GATCGTTGCCGAGATGCCGACTAAGAAATTGTGGTCGTACGACATCA*<br>*AGGGTCCGGCAAAGATTGAGAACAAAAAAGTGTGGGGTCACATTCC*<br>*GGGTACGCATGAGGGTGGCGCGGATGGTATGGATTTTGACGAAGAT*<br>*AACAACTTGCTGGTTGCAAATTGGGGTTCTAGCCACATCGAAGTCTT*<br>*TGGTCCGGATGGTGGCCAGCCGAAGATGCGCATCCGTTGCCCGTTCG*<br>*AGAAGCCGAGCAATCTGCACTTCAAGCCGCAGACCAAGACGATTTT*<br>*TGTGACCGAGCATGAGAACAATGCAGTTTGGAAATTTGAGTGGCAA*<br>*CGTAACGGTAAGAAACAGTATTGTGAAACCCTGAAGTTTGGTATCTT*<br>*C****CACCACCACCACCACCAC***TGA
L34- DFPase	TTTGTTTAACTTTAAGAAGGAGATATACCATGAAAGCGACCAAA<br>CTGGTGCTGGGCGCGGTGATTCTGGGCAGCACCCTGCTGGCGGG<br>C**TGCAGCAGCAACGCGAAAATTGATCA**GGGCGGTGGCAGCGG<br>CGGTGGCAGCGGCGGTGGGAGCGCCACCGGTCCGGCAAGCGGT<br>CCGACCAGCGCAGGTCCGCCGGCATCTCCGGCCCCGCCGGCTGG<br>TCCAGCACCGCCGGCCCCAACCGCACCG*ATGGGGGAAATCCCTGT*<br>*CATTGAGCCGCTGTTTACCAAAGTCACCGAAGATATCCCGGGTGCCG*<br>*AGGGTCCGGTGTTCGACAAAAACGGTGACTTTTACATCGTTGCGCCG*<br>*GCAGTTGAAGTGAATGGCAAGCCTGCTGGTGAGATTCTGCGTATTGA*<br>*CCTGAAAACGGGCAAGAAAACGGTGATTTGTAAACCTGAAGTTAAT*<br>*GGTTATGGCGGCATCCCGGCCGGTTGCCAATGCGATCGTGATGCCAA*<br>*CCAACTGTTCGTGGCAGACATGCGCCTGGGCCTGTTGGTCGTTCAGA*<br>*CCGACGGCACCTTCGAAGAAATCGCGAAAAAAGACTCCGAGGGTCG*<br>*TCGTATGCAGGGCTGCAATGACTGCGCGTTCGACTACGAGGGCAATC*<br>*TGTGGATTACCGCGCCAGCGGGCGAAGTCGCTCCGGCGGATGCTAC*<br>*CGCGAGCATGCAAGAAAAGTTCGGTAGCATTTATTGTTTCACGACTG*<br>*ATGGCCAGATGATTCAAGTCGATACGGCGTTCCAATTTCCGAATGGT*<br>*ATTGCGGTCCGTCACATGAACGATGGTCGCCCGTACCAGCTGATCGT*<br>*TGCCGAGATGCCGACTAAGAAATTGTGGTCGTACGACATCAAGGGT*<br>*CCGGCAAAGATTGAGAACAAAAAAGTGTGGGGTCACATTCCGGGTA*<br>*CGCATGAGGGTGGCGCGGATGGTATGGATTTTGACGAAGATAACAA*<br>*CTTGCTGGTTGCAAATTGGGGTTCTAGCCACATCGAAGTCTTTGGTC*<br>*CGGATGGTGGCCAGCCGAAGATGCGCATCCGTTGCCCGTTCGAGAA*<br>*GCCGAGCAATCTGCACTTCAAGCCGCAGACCAAGACGATTTTTGTGA*

	CCGAGCATGAGAACAATGCAGTTTGGAAATTTGAGTGGCAACGTAA<br>*CGGTAAGAAACAGTATTGTGAAACCCTGAAGTTTGGTATCTTC* ***CACC***<br>***ACCACCACCACCAC*** TGA
DFPase -SC	ATGAACAATAACGATCTCTTTCAGGCATCACGTCGGCGTTTTCTG<br>GCACAACTCGGCGGCTTAACCGTCGCCGGGATGCTGGGGCCGTC<br>ATTGTTAACGCCGCGACGTGCGACTGCGGCGCAAGCGCGGGGTT<br>CTCATCATCATCATCATCAT *ATGGGGGAAATCCCTGTCATTGAGCC*<br>*GCTGTTTACCAAAGTCACCGAAGATATCCCGGGTGCCGAGGGTCCG*<br>*GTGTTCGACAAAAACGGTGACTTTTACATCGTTGCGCCGGCAGTTGA*<br>*AGTGAATGGCAAGCCTGCTGGTGAGATTCTGCGTATTGACCTGAAAA*<br>*CGGGCAAGAAAACGGTGATTTGTAAACCTGAAGTTAATGGTTATGG*<br>*CGGCATCCCGGCCGGTTGCCAATGCGATCGTGATGCCAACCAACTGT*<br>*TCGTGGCAGACATGCGCCTGGGCCTGTTGGTCGTTCAGACCGACGGC*<br>*ACCTTCGAAGAAATCGCGAAAAAAGACTCCGAGGGTCGTCGTATGC*<br>*AGGGCTGCAATGACTGCGCGTTCGACTACGAGGGCAATCTGTGGATT*<br>*ACCGCGCCAGCGGGCGAAGTCGCTCCGGCGGATGCTACCGCGAGCA*<br>*TGCAAGAAAAGTTCGGTAGCATTTATTGTTTCACGACTGATGGCCAG*<br>*ATGATTCAAGTCGATACGGCGTTCCAATTTCCGAATGGTATTGCGGT*<br>*CCGTCACATGAACGATGGTCGCCCGTACCAGCTGATCGTTGCCGAGA*<br>*TGCCGACTAAGAAATTGTGGTCGTACGACATCAAGGGTCCGGCAAA*<br>*GATTGAGAACAAAAAAGTGTGGGGTCACATTCCGGGTACGCATGAG*<br>*GGTGGCGCGGATGGTATGGATTTTGACGAAGATAACAACTTGCTGGT*<br>*TGCAAATTGGGGTTCTAGCCACATCGAAGTCTTTGGTCCGGATGGTG*<br>*GCCAGCCGAAGATGCGCATCCGTTGCCCGTTCGAGAAGCCGAGCAA*<br>*TCTGCACTTCAAGCCGCAGACCAAGACGATTTTTGTGACCGAGCATG*<br>*AGAACAATGCAGTTTGGAAATTTGAGTGGCAACGTAACGGTAAGAA*<br>*ACAGTATTGTGAAACCCTGAAGTTTGGTATCTTCG* GTACCGGTGGC<br>AGCGTTGATACCTTATCAGGTTTATCAAGTGAGCAAGGTCAGTCC<br>GGTGATATGACAATTGAAGAAGATAGTGCTACCCATATTAAATT<br>CTCAAAACGTGATGAGGACGGCAAAGAGTTAGCTGGTGCAACTA<br>TGGAGTTGCGTGATTCATCTGGTAAAACTATTAGTACATGGATTT<br>CAGATGGACAAGTGAAAGATTTCTACCTGTATCCAGGAAAATAT<br>ACATTTGTCGAAACCGCAGCACCAGACGGTTATGAGGTAGCAAC<br>TGCTATTACCTTTACAGTTAATGAGCAAGGTCAGGTTACTGTAAA<br>TGGCAAAGCAACTAAAGGTGACGCTCATATTAGCGGAGGTGGA<br>GGTGAGCTCGTCGACTAA
BtuF-DFPase	ATGGCTAAGTCACTGTTCAGGGCGCTGGTCGCCCTGTCTTTTCTTG<br>CGCCACTGTGGCTCAACGCCGCGCCGCGCGTCATCACGCTTTCTC<br>CCGCCAACACTGAACTTGCCTTTGCCGCCGGGATCACGCCGGTTG<br>GGGTCAGCAGCTATTCCGACTATCCTCCACAAGCGCAAAAGATT<br>GAGCAGGTTTCCACCTGGCAGGGGATGAATCTGGAACGCATTGT<br>CGCGCTGAAACCCGATCTGGTGATTGCCTGGCGTGGAGGTAATG<br>CCGAGCGGCAGGTTGACCAGCTGGCTTCGCTGGGAATAAAAGTG<br>ATGTGGGTCGATGCGACAAGCATTGAACAAATTGCCAATGCGTT<br>ACGTCAACTGGCCCCCTGGAGTCCGCAACCAGACAAGGCCGAA<br>CAAGCCGCGCAATCCCTGCTGGATCAGTACGCGCAATTGAAAGC<br>GCAATATGCTGATAAACCTAAAAAACGTGTTTTTCTGCAATTCGG<br>CATTAATCCGCCATTTACCAGTGGAAAAGAGTCGATTCAGAACC<br>AGGTACTCGAAGTTTGTGGCGGAGAAAACATCTTTAAAGACAGC

	CGGGTTCCCTGGCCGCAAGTTAGCCGCGAACAGGTGTTAGCACG<br>CTCGCCACAGGCGATTGTCATTACAGGCGGACCGGACCAAATTC<br>CTAAAATCAAACAATACTGGGGTGAACAGCTCAAAATTCCCGTT<br>ATTCCTCTCACGAGTGACTGGTTTGAACGTGCAAGCCCACGTATT<br>ATCCTCGCTGCACAACAGCTCTGTAATGCGCTTTCACAGGTAGAT<br>AATTCGAGCTCGAACAACAACAACAATAACAATAACAACAACC<br>TCGGGATCGAGGGAAGGATTTCACATATGAGCATTGGCACCGGC<br>GAT*ATGGGGGAAATCCCTGTCATTGAGCCGCTGTTTACCAAAGTCAC*<br>*CGAAGATATCCCGGGTGCCGAGGGTCCGGTGTTCGACAAAAACGGT*<br>*GACTTTTACATCGTTGCGCCGGCAGTTGAAGTGAATGGCAAGCCTGC*<br>*TGGTGAGATTCTGCGTATTGACCTGAAAACGGGCAAGAAAACGGTG*<br>*ATTTGTAAACCTGAAGTTAATGGTTATGGCGGCATCCCGGCCGGTTG*<br>*CCAATGCGATCGTGATGCCAACCAACTGTTCGTGGCAGACATGCGCC*<br>*TGGGCCTGTTGGTCGTTCAGACCGACGGCACCTTCGAAGAAATCGCG*<br>*AAAAAAGACTCCGAGGGTCGTCGTATGCAGGGCTGCAATGACTGCG*<br>*CGTTCGACTACGAGGGCAATCTGTGGATTACCGCGCCAGCGGGCGA*<br>*AGTCGCTCCGGCGGATGCTACCGCGAGCATGCAAGAAAAGTTCGGT*<br>*AGCATTTATTGTTTCACGACTGATGGCCAGATGATTCAAGTCGATAC*<br>*GGCGTTCCAATTTCCGAATGGTATTGCGGTCCGTCACATGAACGATG*<br>*GTCGCCCGTACCAGCTGATCGTTGCCGAGATGCCGACTAAGAAATTG*<br>*TGGTCGTACGACATCAAGGGTCCGGCAAAGATTGAGAACAAAAAAG*<br>*TGTGGGGTCACATTCCGGGTACGCATGAGGGTGGCGCGGATGGTAT*<br>*GGATTTTGACGAAGATAACAACTTGCTGGTTGCAAATTGGGGTTCTA*<br>*GCCACATCGAAGTCTTTGGTCCGGATGGTGGCCAGCCGAAGATGCG*<br>*CATCCGTTGCCCGTTCGAGAAGCCGAGCAATCTGCACTTCAAGCCGC*<br>*AGACCAAGACGATTTTTGTGACCGAGCATGAGAACAATGCAGTTTG*<br>*GAAATTTGAGTGGCAACGTAACGGTAAGAAACAGTATTGTGAAACC*<br>*CTGAAGTTTGGTATCTTC*GAATTCCCTGCAGGTAATTAA
MBP- DFPase	ATGAAAATAAAAACAGGTGCACGCATCCTCGCATTATCCGCATT<br>AACGACGATGATGTTTTCCGCCTCGGCTCTCGCCAAAATCGAAG<br>AAGGTAAACTGGTAATCTGGATTAACGGCGATAAAGGCTATAAC<br>GGTCTCGCTGAAGTCGGTAAGAAATTCGAGAAAGATACCGGAAT<br>TAAAGTCACCGTTGAGCATCCGGATAAACTGGAAGAGAAATTCC<br>CACAGGTTGCGGCAACTGGCGATGGCCCTGACATTATCTTCTGGG<br>CACACGACCGCTTTGGTGGCTACGCTCAATCTGGCCTGTTGGCTG<br>AAATCACCCCGGACAAAGCGTTCCAGGACAAGCTGTATCCGTTT<br>ACCTGGGATGCCGTACGTTACAACGGCAAGCTGATTGCTTACCC<br>GATCGCTGTTGAAGCGTTATCGCTGATTTATAACAAAGATCTGCT<br>GCCGAACCCGCCAAAAACCTGGGAAGAGATCCCGGCGCTGGAT<br>AAAGAACTGAAAGCGAAAGGTAAGAGCGCGCTGATGTTCAACC<br>TGCAAGAACCGTACTTCACCTGGCCGCTGATTGCTGCTGACGGG<br>GGTTATGCGTTCAAGTATGAAAACGGCAAGTACGACATTAAAGA<br>CGTGGGCGTGGATAACGCTGGCGCGAAAGCGGGTCTGACCTTCC<br>TGGTTGACCTGATTAAAAACAAACACATGAATGCAGACACCGAT<br>TACTCCATCGCAGAAGCTGCCTTTAATAAAGGCGAAACAGCGAT<br>GACCATCAACGGCCCGTGGGCATGGTCCAACATCGACACCAGCA<br>AAGTGAATTATGGTGTAACGGTACTGCCGACCTTCAAGGGTCAA<br>CCATCCAAACCGTTCGTTGGCGTGCTGAGCGCAGGTATTAACGC<br>CGCCAGTCCGAACAAAGAGCTGGCAAAAGAGTTCCTCGAAAAC<br>TATCTGCTGACTGATGAAGGTCTGGAAGCGGTTAATAAAGACAA

	ACCGCTGGGTGCCGTAGCGCTGAAGTCTTACGAGGAAGAGTTGG<br>TGAAAGATCCGCGTATTGCCGCCACTATGGAAAACGCCCAGAAA<br>GGTGAAATCATGCCGAACATCCCGCAGATGTCCGCTTTCTGGTAT<br>GCCGTGCGTACTGCGGTGATCAACGCCGCCAGCGGTCGTCAGAC<br>TGTCGATGAAGCCCTGAAAGACGCGCAGACTAATTCGAGCTCGA<br>ACAACAACAACAATAACAATAACAACAACCTCGGGATCGAGGG<br>AAGGATTTCACATATGAGCATTGGCACCGGCGAT*ATGGGGGAAA*<br>*TCCCTGTCATTGAGCCGCTGTTTACCAAAGTCACCGAAGATATCCCG*<br>*GGTGCCGAGGGTCCGGTGTTCGACAAAAACGGTGACTTTTACATCGT*<br>*TGCGCCGGCAGTTGAAGTGAATGGCAAGCCTGCTGGTGAGATTCTGC*<br>*GTATTGACCTGAAAACGGGCAAGAAAACGGTGATTTGTAAACCTGA*<br>*AGTTAATGGTTATGGCGGCATCCCGGCCGGTTGCCAATGCGATCGTG*<br>*ATGCCAACCAACTGTTCGTGGCAGACATGCGCCTGGGCCTGTTGGTC*<br>*GTTCAGACCGACGGCACCTTCGAAGAAATCGCGAAAAAAGACTCCG*<br>*AGGGTCGTCGTATGCAGGGCTGCAATGACTGCGCGTTCGACTACGA*<br>*GGGCAATCTGTGGATTACCGCGCCAGCGGGCGAAGTCGCTCCGGCG*<br>*GATGCTACCGCGAGCATGCAAGAAAAGTTCGGTAGCATTTATTGTTT*<br>*CACGACTGATGGCCAGATGATTCAAGTCGATACGGCGTTCCAATTTC*<br>*CGAATGGTATTGCGGTCCGTCACATGAACGATGGTCGCCCGTACCAG*<br>*CTGATCGTTGCCGAGATGCCGACTAAGAAATTGTGGTCGTACGACAT*<br>*CAAGGGTCCGGCAAAGATTGAGAACAAAAAAGTGTGGGGTCACATT*<br>*CCGGGTACGCATGAGGGTGGCGCGGATGGTATGGATTTTGACGAAG*<br>*ATAACAACTTGCTGGTTGCAAATTGGGGTTCTAGCCACATCGAAGTC*<br>*TTTGGTCCGGATGGTGGCCAGCCGAAGATGCGCATCCGTTGCCCGTT*<br>*CGAGAAGCCGAGCAATCTGCACTTCAAGCCGCAGACCAAGACGATT*<br>*TTTGTGACCGAGCATGAGAACAATGCAGTTTGGAAATTTGAGTGGCA*<br>*ACGTAACGGTAAGAAACAGTATTGTGAAACCCTGAAGTTTGGTATCT*<br>*TC*GAATTCCCTGCAGGTAATTAA
SLP- DFPaseE	ATGAACATGACAAAAGGTGCACTCATCCTCAGCCTTTCATTTTTG<br>CTTGCCGCATGTAGTTCAATTCCGCAAAATATCAAAGGCAATAA<br>CCAACCTGATATTCAAAAAAGTTTTGTTGCTGTTCATAACCAGCC<br>GGGGTTATATGTTGGTCAACAAGCGCGCTTTGGTGGGAAGGTTAT<br>CAACGTTATCAATGGCAAAACGGATACGTTGTTAGAAATCTCTGT<br>ATTACCGTTGGATAGCTATGCGAAGCCTGATATTGAAGCCAACT<br>ATCAGGGCCGACTGCTCGCCAGACAAAGCGGCTTCCTTGATCCA<br>GTGAACTATCGTAATCACTTTGTTACCATCCTCGGCACCATTCAG<br>GGTGAACAACCTGGCTTTATCAATAAAGTCCCGTATAACTTCCTG<br>GAAGTGAATATGCAGGGCATCCAGGTGTGGCATTTGAGAGAAGT<br>GGTTAATACCACCTATAACCTGTGGGATTACGGCTATGGTGCATT<br>CTGGCCGGAACCGGGCTGGGGTGCGCCTTACTACACCAATGCGG<br>TGAGTCAGGTAACACCTGAGCTGGTCAAAAATTCGAGCTCGAAC<br>AACAACAACAATAACAATAACAACAACCTCGGGATCGAGGGAA<br>GGATTTCACATATGAGCATTGGCACCGGCGAT*ATGGGGGAAATCC*<br>*CTGTCATTGAGCCGCTGTTTACCAAAGTCACCGAAGATATCCCGGGT*<br>*GCCGAGGGTCCGGTGTTCGACAAAAACGGTGACTTTTACATCGTTGC*<br>*GCCGGCAGTTGAAGTGAATGGCAAGCCTGCTGGTGAGATTCTGCGT*<br>*ATTGACCTGAAAACGGGCAAGAAAACGGTGATTTGTAAACCTGAAG*<br>*TTAATGGTTATGGCGGCATCCCGGCCGGTTGCCAATGCGATCGTGAT*<br>*GCCAACCAACTGTTCGTGGCAGACATGCGCCTGGGCCTGTTGGTCGT*<br>*TCAGACCGACGGCACCTTCGAAGAAATCGCGAAAAAAGACTCCGAG*

GGTCGTCGTATGCAGGGCTGCAATGACTGCGCGTTCGACTACGAGG
GCAATCTGTGGATTACCGCGCCAGCGGGCGAAGTCGCTCCGGCGGA
TGCTACCGCGAGCATGCAAGAAAAGTTCGGTAGCATTTATTGTTTCA
CGACTGATGGCCAGATGATTCAAGTCGATACGGCGTTCCAATTTCCG
AATGGTATTGCGGTCCGTCACATGAACGATGGTCGCCCGTACCAGCT
GATCGTTGCCGAGATGCCGACTAAGAAATTGTGGTCGTACGACATCA
AGGGTCCGGCAAAGATTGAGAACAAAAAAGTGTGGGGTCACATTCC
GGGTACGCATGAGGGTGGCGCGGATGGTATGGATTTTGACGAAGAT
AACAACTTGCTGGTTGCAAATTGGGGTTCTAGCCACATCGAAGTCTT
TGGTCCGGATGGTGGCCAGCCGAAGATGCGCATCCGTTGCCCGTTCG
AGAAGCCGAGCAATCTGCACTTCAAGCCGCAGACCAAGACGATTTT
TGTGACCGAGCATGAGAACAATGCAGTTTGGAAATTTGAGTGGCAA
CGTAACGGTAAGAAACAGTATTGTGAAACCCTGAAGTTTGGTATCTT
*C*GAATTCCCTGCAGGTAATTAA

SlyB- DFPase

ATGATTAAACGCGTATTGGTTGTTTCAATGGTAGGTCTGTCTCTTG
TCGGTTGTGTTAATAACGACACCCTGTCAGGGGATGTTTATACCG
CTTCTGAAGCGAAACAAGTACAGAATGTCAGCTATGGCACCATC
GTTAACGTACGTCCGGTACAGATTCAGGGCGGTGATGATTCCAA
CGTTATCGGTGCAATTGGCGGTGCTGTTCTTGGTGGTTTCCTGGGG
AATACTGTTGGTGGCGGAACCGGGCGTTCTCTGGCTACTGCAGC
AGGCGCTGTTGCAGGTGGCGTAGCTGGTCAGGGCGTACAGAGTG
CAATGAACAAAACGCAGGGTGTCGAGCTGGAAATTCGTAAAGA
CGATGGTAATACCATCATGGTGGTACAGAAACAAGGCAACACTC
GTTTCTCTCCGGGCCAACGTGTCGTACTGGCCAGCAATGGCAGTC
AGGTGACCGTTTCTCCGCGCAATTCGAGCTCGAACAACAACAAC
AATAACAATAACAACAACCTCGGGATCGAGGGAAGGATTTCAC
ATATGAGCATTGGCACCGGCGAT*ATGGGGGAAATCCCTGTCATTG*
AGCCGCTGTTTACCAAAGTCACCGAAGATATCCCGGGTGCCGAGGG
TCCGGTGTTCGACAAAAACGGTGACTTTTACATCGTTGCGCCGGCAG
TTGAAGTGAATGGCAAGCCTGCTGGTGAGATTCTGCGTATTGACCTG
AAAACGGGCAAGAAAACGGTGATTTGTAAACCTGAAGTTAATGGTT
ATGGCGGCATCCCGGCCGGTTGCCAATGCGATCGTGATGCCAACCA
ACTGTTCGTGGCAGACATGCGCCTGGGCCTGTTGGTCGTTCAGACCG
ACGGCACCTTCGAAGAAATCGCGAAAAAAGACTCCGAGGGTCGTCG
TATGCAGGGCTGCAATGACTGCGCGTTCGACTACGAGGGCAATCTGT
GGATTACCGCGCCAGCGGGCGAAGTCGCTCCGGCGGATGCTACCGC
GAGCATGCAAGAAAAGTTCGGTAGCATTTATTGTTTCACGACTGATG
GCCAGATGATTCAAGTCGATACGGCGTTCCAATTTCCGAATGGTATT
GCGGTCCGTCACATGAACGATGGTCGCCCGTACCAGCTGATCGTTGC
CGAGATGCCGACTAAGAAATTGTGGTCGTACGACATCAAGGGTCCG
GCAAAGATTGAGAACAAAAAAGTGTGGGGTCACATTCCGGGTACGC
ATGAGGGTGGCGCGGATGGTATGGATTTTGACGAAGATAACAACTT
GCTGGTTGCAAATTGGGGTTCTAGCCACATCGAAGTCTTTGGTCCGG
ATGGTGGCCAGCCGAAGATGCGCATCCGTTGCCCGTTCGAGAAGCC
GAGCAATCTGCACTTCAAGCCGCAGACCAAGACGATTTTTGTGACCG
AGCATGAGAACAATGCAGTTTGGAAATTTGAGTGGCAACGTAACGG
*TAAGAAACAGTATTGTGAAACCCTGAAGTTTGGTATCTTC*GAATTCC
CTGCAGGTAATTAA

RBS – Underlined.

Lpp' – Bold

Linker/Fusion partner - Shaded

6x-His – Bold, Italics

PTE/DFPase – Italics, Underlined
